# An Integrated Cell Purification and Genomics Strategy Reveals Multiple Regulators of Pancreas Development

**DOI:** 10.1371/journal.pgen.1004645

**Published:** 2014-10-16

**Authors:** Cecil M. Benitez, Kun Qu, Takuya Sugiyama, Philip T. Pauerstein, Yinghua Liu, Jennifer Tsai, Xueying Gu, Amar Ghodasara, H. Efsun Arda, Jiajing Zhang, Joseph D. Dekker, Haley O. Tucker, Howard Y. Chang, Seung K. Kim

**Affiliations:** 1 Department of Developmental Biology, Stanford University School of Medicine, Stanford, California, United States of America; 2 Program in Epithelial Biology, Stanford University School of Medicine, Stanford, California, United States of America; 3 Institute for Cellular and Molecular Biology and Department of Molecular Biosciences, University of Texas at Austin, Austin, Texas, United States of America; 4 Department of Dermatology, Stanford University School of Medicine,, Stanford, California, United States of America; 5 Howard Hughes Medical Institute, Stanford University School of Medicine, Stanford, California, United States of America; 6 Department of Medicine (Oncology Division), Stanford University School of Medicine, Stanford, California, United States of America; University of Wisconsin-Madison, United States of America

## Abstract

The regulatory logic underlying global transcriptional programs controlling development of visceral organs like the pancreas remains undiscovered. Here, we profiled gene expression in 12 purified populations of fetal and adult pancreatic epithelial cells representing crucial progenitor cell subsets, and their endocrine or exocrine progeny. Using probabilistic models to decode the general programs organizing gene expression, we identified co-expressed gene sets in cell subsets that revealed patterns and processes governing progenitor cell development, lineage specification, and endocrine cell maturation. Purification of *Neurog3* mutant cells and module network analysis linked established regulators such as *Neurog3* to unrecognized gene targets and roles in pancreas development. Iterative module network analysis nominated and prioritized transcriptional regulators, including diabetes risk genes. Functional validation of a subset of candidate regulators with corresponding mutant mice revealed that the transcription factors *Etv1*, *Prdm16*, *Runx1t1* and *Bcl11a* are essential for pancreas development. Our integrated approach provides a unique framework for identifying regulatory genes and functional gene sets underlying pancreas development and associated diseases such as diabetes mellitus.

## Author Summary

Discovery of specific pancreas developmental regulators has accelerated in recent years. In contrast, the global regulatory programs controlling pancreas development are poorly understood compared to other organs or tissues like heart or blood. Decoding this regulatory logic may accelerate development of replacement organs from renewable sources like stem cells, but this goal requires identification of regulators and assessment of their functions on a global scale. To address this important challenge for pancreas biology, we combined purification of normal and mutant cells with genome-scale methods to generate and analyze expression profiles from developing pancreas cells. Our work revealed regulatory gene sets governing development of pancreas progenitor cells and their progeny. Our integrative approach nominated multiple pancreas developmental regulators, including suspected risk genes for human diabetes, which we validated by phenotyping mutant mice on a scale not previously reported. Selection of these candidate regulators was unbiased; thus it is remarkable that all were essential for pancreatic islet development. Thus, our studies provide a new heuristic resource for identifying genetic functions underlying pancreas development and diseases like diabetes mellitus.

## Introduction

The pancreas is a vital internal organ with exocrine and endocrine functions. The exocrine pancreas is composed of acinar cells that secrete digestive enzymes into a branched network of bicarbonate-secreting duct cells. Endocrine cells form clusters called islets of Langerhans that secrete hormones such as insulin, glucagon, pancreatic polypeptide, somatostatin, and ghrelin produced, respectively, by beta cells, alpha cells, PP cells, delta cells and a transient population of epsilon cells [1]. Classical genetic approaches revealed that exocrine and endocrine cells develop from a common multipotent progenitor that expresses the transcription factors *Sox9* [2] [3], *Pdx1* [4], and *Ptf1a* [5]. Through mouse embryonic development, Sox9^+^ multipotent progenitors generate endocrine progenitors that express the basic helix-loop-helix (bHLH) transcription factor *Neurog3* [6], which produce all pancreatic endocrine cells [7]. Although these approaches have revealed much about individual factors that regulate pancreatic development [8], we have yet to understand the regulatory logic underlying pancreas formation [9].

Genome-scale approaches to organ development can provide unbiased views of the genetic interactions regulating transient cell populations such as multipotent progenitor cells and their lineage-restricted progeny. However, to decipher the regulatory logic that culminates in successful organogenesis, gene expression in distinct, developing cell types must be acquired. Then, algorithms that analyze expression patterns across multiple cell types can be executed. Such integrated efforts have been used successfully for studies of hematopoiesis [10] [11]. We postulate that similar approaches to identify gene sets orchestrating formation and maturation of pancreas cells should advance islet beta cell-replacement efforts in diabetic patients. However, the application of such an approach to solid organs like the pancreas has proven difficult. In particular, crucial transient cell subsets like multipotent pancreatic progenitors and endocrine precursors represent a small fraction of cells in the developing pancreas – making analysis of these cells challenging. Thus, while prior studies used cell fractionation and genome-scale analysis of gene expression to advance understanding of pancreas development [12] [13] [14] [15] none was able to comprehensively assess purified progenitors and their endocrine or exocrine progeny from multiple developmental stages. Moreover, most previous studies were limited to pair-wise comparisons of wild-type cells in the endocrine lineage, thereby precluding powerful inferences from mutant analysis, and none prioritized or validated multiple candidate regulators by phenotyping mutant mice.

Here, we used a combination of cell sorting and transgenic cell labeling to purify and profile twelve pancreatic cell types at specific stages of development and used methods to optimize RNA quantification from relatively small numbers of cells. Our data set encompasses multipotent Sox9^+^ pancreatic progenitors, Neurog3^+^ endocrine progenitors, fetal and adult alpha cells and beta cells, and exocrine cells including fetal acinar cells and adult duct cells. Statistical comparisons demonstrate the highly reproducible quality of gene expression profiles obtained. We found that iterative probabilistic modeling, optimized with data on established pancreatic regulators, succeeded in nominating and ranking scores of novel candidate regulators and their functions. We validated a subset of these predictions using mutant *Neurog3* mice and by phenotyping pancreas development in appropriate mutant mice. This comprehensive, integrated effort with discrete, operationally-defined populations of purified fetal and adult pancreatic cells provides gene expression profiling at higher resolution than previously achieved, identifies new regulators of pancreas development that are validated in vivo, and elucidates new elements of the regulatory logic underlying development of the endocrine and exocrine pancreas.

## Results

### Purification and gene expression profiling of fetal and adult pancreatic cells

**Figure 1:**
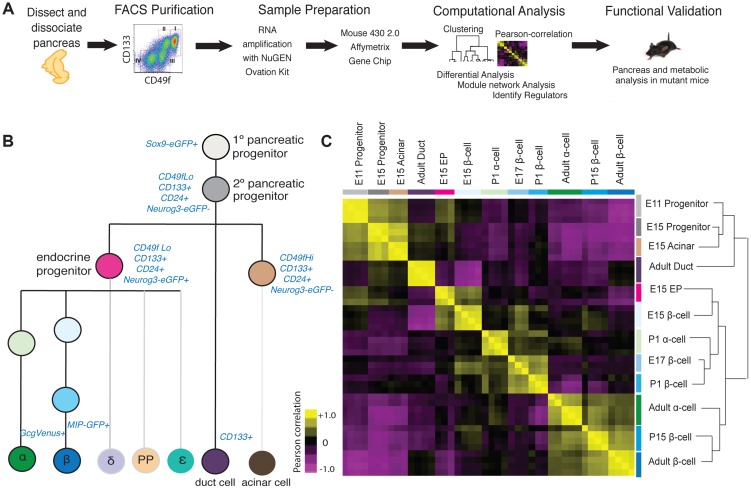
Acquisition and analysis of global gene-expression. (A) Schematic of experiments in this study. (B) Lineage-diagram of pancreas development. The following cell types were collected: E11 and E15 pancreatic progenitors, E15 acinar cells, E15 endocrine progenitors (EP), E15, E17, P1, P15, 8–12 week beta cells, P1 and 8–12 week alpha cells, and adult duct cells. The sort strategy is displayed in blue. Each sample was collected in at least triplicate. MIP: Mouse Insulin Promoter, GcgVenus: Glucagon-Venus. (C) Pearson correlation plot and hierarchical clustering (right) of 12 cell populations. The Pearson correlation coefficient was calculated on mean-centered normalized expression values of a subset of significant expressed genes (see Methods for details). A positive correlation is portrayed in yellow and a negative correlation in purple.

To dissect developmental mechanisms of pancreatic development and maturation, we adopted a strategy using staged mice, FACS purification of specific cell subsets, genome-scale gene expression profiling coupled to bioinformatics analysis, and validation using mutant mice (Figure 1A). Using a combination of surface markers and transgenic reporter mice, we isolated 12 cell populations and profiled gene expression using GeneChip microarrays (Figure 1B; Methods). These included embryonic day (E) 11 cells enriched for Sox9^+^ multipotent pancreatic progenitors [3], E15 pancreatic ‘progenitors’ enriched for the markers Sox9 and CD24 [16] [17], E15 Neurog3^+^ endocrine progenitors enriched for CD133 and CD49f [16], E15 acinar cells, Glucagon^+^ alpha cells from postnatal day (P) 1 and 8–12 weeks, fetal and adult beta cells from E15, E17, P1, P15, and 8–12 weeks, and duct cells from 8–12 weeks. To our knowledge, comparative analysis of this range of mouse pancreatic cell types and developmental stages has not been reported.

To assess the quality and reproducibility of replicate cell isolations, RNA collection and gene expression profiles, we obtained the Pearson correlation coefficient of pairwise-comparisons between samples and performed unsupervised hierarchal clustering. This analysis revealed tight clustering of biological replicates for each cell subset isolated (Figure 1C). We verified the expression of established pancreatic markers and developmental regulators [9] for each specific cell type profiled, using microarray (Figure S1A), or with quantitative PCR for a subset of ultra-abundant mRNAs encoding proteins like insulin and glucagon, which can saturate microarray probes (Figure S1B, S1C). Sox9^+^ E11 and E15 pancreatic progenitor cells were enriched for expression of expected mRNAs encoding *Sox9*, *cMyc*, and *Onecut1*. Likewise we confirmed that adult ductal cells were enriched for expression of *Muc1*, *Sox9*, *Onecut1* and *Hes1* mRNA (Figure S1A). At multiple stages, purified beta cells were highly enriched for mRNAs encoding *Pdx1, Insulin* and *Glucokinase*, while alpha cells expressed expected markers, including *Pax6*, *MafB*, *Arx*, and *Glucagon* (Figure S1A). Thus, appropriate scaling of mouse collections overcame inherently low numbers of fetal pancreatic cell subsets to generate a unique, coherent set of highly reproducible gene expression data sets spanning multiple pancreatic cell lineages and developmental stages.

Pearson correlation and unsupervised hierarchical clustering analysis revealed grouping of cell types and their gene expression based on developmental stage, and exocrine or endocrine function. Undifferentiated fetal pancreatic progenitors from E11 clustered closest to E15 progenitor cells and E15 acinar cells (Figure 1C). E15 Neurog3^+^ endocrine progenitors clustered closely with fetal alpha cells and beta cells, forming a cluster distinct from fetal progenitor, ductal and acinar cells. Adult duct cell gene expression clustered with that of E11 and E15 pancreatic progenitors cells, instead of other adult cells, likely reflecting the postulated origins of pancreatic progenitors from primitive fetal ductal cells [18]. Unexpectedly, we did not observe clustering by endocrine cell type; rather, we observed clustering of postnatal and adult beta cells with adult alpha cells, and close clustering of fetal beta cells with neonatal alpha cells (Figure 1C). Pair-wise differential expression analysis (Table S1) and unsupervised hierarchical clustering analysis with over 30 adult mouse tissues [19] supported this conclusion (Figure S2). As described below, this similarity likely reflects common functions of mature adult alpha cells and beta cells as nutrient-responsive cells that produce, process and secrete peptide hormones—functions distinct from those in fetal alpha and beta cells.

### Identifying distinct gene sets in pancreas progenitor and exocrine development

**Figure 2:**
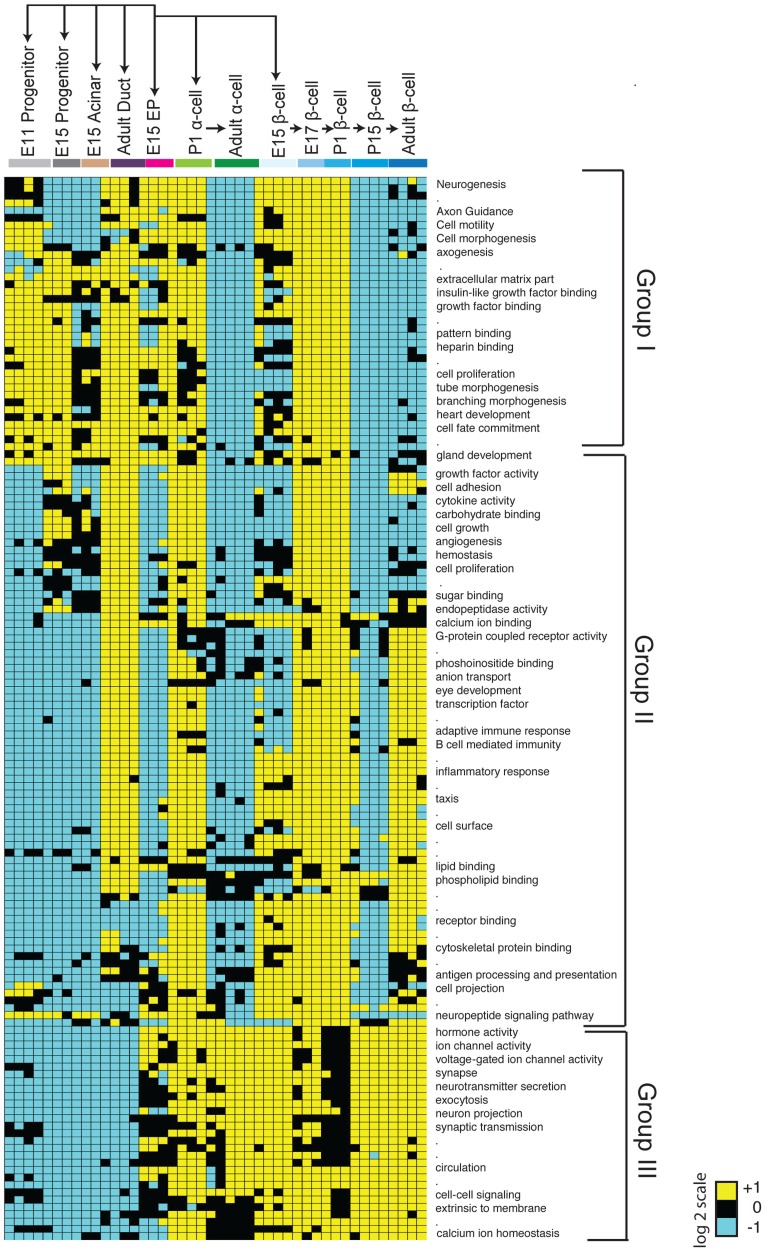
Module map analysis of differentially expressed gene sets. The module map algorithm of Genomica software was executed to identify gene-sets (representing gene ontology biological functions) that are differentially expressed between 12 cell populations representing various stages and cell types of the pancreas. Each individual block represents the average expression of statistically enriched (yellow) or depleted (teal) genes based on a log2 scale (*P*<0.05 and FDR<0.05, Cut-off values >1 or <−1, based on a log2 scale). Black blocks indicate that there was no significant enrichment or depletion of a gene-set. Because of resolution and space constraints not all gene set terms are displayed (signified with dots). Endocrine progenitor (EP).

The clustering of cell types in our Pearson correlation analysis (Figure 1C) indicated that specific cell types expressed distinct genes. To investigate this further, we used module mapping [20], which determines if a set of genes associated with or governing a specific biological function is significantly enriched or depleted within a sample (see Methods). As expected, module mapping revealed gene sets enriched in E11 pancreatic progenitors that remained enriched in E15 pancreatic progenitors, fetal acinar cells, and duct cells (Group I; Figure 2). This included gene sets regulating cell proliferation, cell fate commitment, branching morphogenesis and gland development; together, these functions reflect the known proliferative capacity and differentiation potential of these cell populations [21]. In addition, we observed that progenitor and duct cells shared modules with fetal endocrine cells (Group I; Figure 2), suggesting common genetic regulatory features. These commonalities are consistent with recent findings indicating a latent potential in pancreatic acinar or duct cells for conversion into endocrine cells [22], [23]. To identify gene sets enriched or uniquely expressed in E11 and E15 Sox9^+^ pancreatic progenitors, acinar or duct cells, we (1) obtained the gene signatures for each cell type (Figure S3; Table S2; see Methods for gene signature criteria), and (2) identified differentially expressed genes (Figure S4; Tables S2, S3, S4). This analysis revealed that E11, E15 progenitors and E15 acinar cells shared distinct but overlapping gene signatures (Figure S3A). Genes in these signatures included many transcriptional regulators, including *Bcl11a*, and genes involved in RNA processing and translation such as *Spin2*, *Rpp40*, and *Rpl23*, whose possible roles have not been previously noted in pancreas development. Thus, our analysis identified genes and gene sets that are expressed during pancreatic progenitor and exocrine cell development.

### Identifying gene sets in endocrine progenitors and their fetal and adult endocrine progeny

Maturation of defining beta cell functions, such as glucose sensing and insulin secretion, increases from fetal through post-natal stages [7] [24]; however, a comprehensive analysis of beta cell gene expression from fetal to adult stages has not, to our knowledge, been reported in mice. Likewise, little is known about gene expression changes accompanying maturation of alpha cells [25]. Module mapping revealed gene sets initially expressed in Sox9^+^ pancreatic progenitor cells and Neurog3^+^ endocrine progenitors (including the terms cell proliferation and cell fate commitment) that were maintained in fetal beta and alpha cells but extinguished in adult endocrine cells (Group I; Figure 2). A second group of gene sets (with terms like cell adhesion, angiogenesis, hormone activity and eye development) was expressed after the Neurog3^+^ stage in alpha and beta cells. Strikingly, nearly all these modules were transiently downregulated in P15 beta cells and lost in adult alpha cells, but maintained in adult beta cells (Group II; Figure 2). These findings are consistent with prior studies showing that at early stages of development (E15, E17, and P1) immature alpha and beta cells are establishing neurovascular connections [26] [27], proliferating [28] [29], and developing components necessary for hormone synthesis, processing or secretion [30]. A third group of modules (associated with terms like voltage-gated ion channel activity, exocytosis, synapse, and calcium ion homeostasis) was expressed initially at the Neurog3^+^ stage then maintained throughout endocrine cell development (Group III; Figure 2). Thus, consistent with the clustering pattern of endocrine cells, we identified many gene sets that were shared between alpha and beta cells.

Next, we sought to identify distinguishing gene sets and signatures between alpha and beta cells. Module mapping revealed that adult beta cells (compared to alpha cells) maintained dozens of distinct gene sets (linked to terms like cell adhesion, calcium ion binding, eye development and G-protein coupled receptor activity; Groups II; Figure 2). These findings are consistent with established roles of GPCRs and calcium transients in regulating adult beta-cell proliferation, maturation and physiological regulation [31]. Our analysis similarly revealed distinct gene signatures between differentiated alpha and beta cells (Figure S3A, S3B; Table S2). Differentially expressed genes enriched in postnatal beta cells included *Cldn8*, *C1qb*, and *Gdf3*, while *Fap*, *Ctxn2*, and *Mctp2* were highly expressed in adult alpha cells (Figure S4; Table S4). Collectively, this work provides a useful resource for exploring gene regulation and development of islet beta and alpha cells (see below).

### Iterative module network analysis (IMNA) identifies regulators of pancreas development

**Figure 3:**
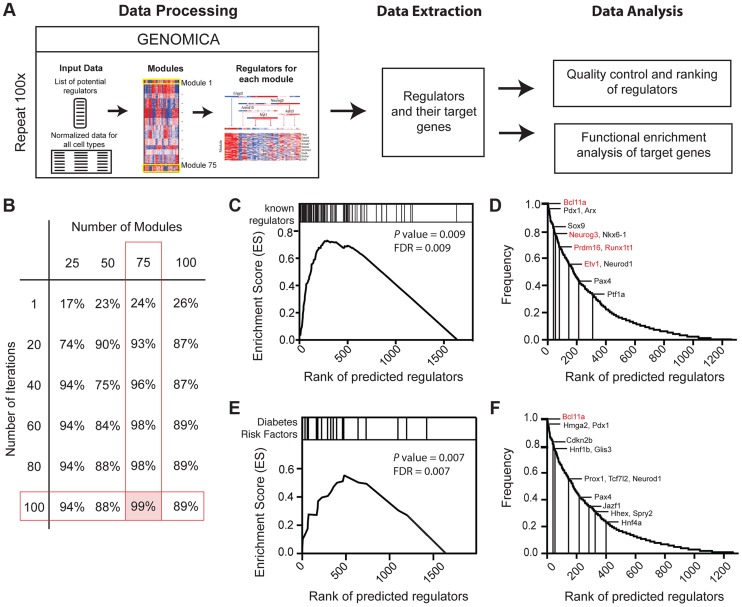
Expression-based identification of pancreatic regulators. (A) Schematic of approach used to identify regulators of pancreas development, their targets, and their predicted biological functions using the module network algorithm of Genomica. To identify regulators two lists are loaded into the program: 1) a list of potential regulators and 2) normalized expression values of samples. Genes with similar expression patterns are grouped (termed a module). Regulators that are most predictive of a specific module expression pattern are learned. Output information includes a list of regulators and their potential targets. Functional enrichment analysis is used to predict the biological function of each regulator (see Methods for details). An example of module-network analysis nominating Neurog3 as a candidate regulator of endocrine development is shown along with its potential targets. (B) Optimal number of modules and iterations were determined by calculating the percentage of known regulators of pancreas development for each module and iteration combination. (C) Gene set enrichment analysis (GSEA) for 100 iterations of 75 modules yielded an enrichment score greater than >0.5 when known regulators were used. Distribution of known regulators based on their rank is displayed on the top panel. (D) Ranking of candidate regulators based on their frequency. Among the most reproducible candidates included known pancreas regulators such as *Pdx1* and *Neurog3* (red font) and candidate regulators validated in subsequent analysis (red font). (E) GSEA plot for the distribution of diabetes risk factors among list of predicted regulators. (F) Ranking of diabetes risk factors based on their frequency score. Validated GWAS genes include Bcl11a (red). (D and F) A frequency of 1.0 means that the candidate regulator appeared in 100% of the iterations performed.

After obtaining genes and gene sets that were differentially expressed during pancreas development, we sought to identify the regulatory logic governing their expression. Mutations in loci encoding transcription factors constitute an important group of risk factors for diabetes mellitus and pancreatic malformations in humans [32] [33]. Thus, we focused on identifying regulatory networks governed by transcription factors. To do this we adapted the module network function in Genomica, a probabilistic algorithm that groups genes based on co-expression patterns (modules) and predicts the regulators that might control such gene co-expression (Figure 3A; [34] [35]). To identify regulators, we first analyzed 1642 genes expressed in the developing pancreas that encode transcription factors (TF) or DNA-binding factors [36] [37] [38]. We optimized our parameters with a ‘training’ set of 82 established pancreatic regulators (Methods; [9]). Because Genomica is constrained to choose one group of regulators per gene set - a recognized limitation [39] - we employed an Iterative Module Network Analysis (IMNA) approach, in which we identified candidate regulators after multiple iterations (‘runs’) of the module network program. We then systematically varied the number of modules and runs (Figure 3B; Figure S5; Methods), and found that 100 iterations of 75 modules identified 99% (81/82) of established transcriptional regulators of pancreas development that included *Neurog3*, *Arx*, *Glis3*, *Pdx1*, *Isl1*, *Fev* and *Myt1* (Figure 3D; Table S5). The quality of predictions did not improve with more iterations or modules (Methods). To determine the validity of our outputs, we ranked candidate regulators based on their frequency of occurrence across all iterations (Figure 3D) and performed Gene Set Enrichment Analysis on these ranked regulators (GSEA; Figure 3C; [40]). This analysis revealed that established pancreatic regulators ranked significantly higher in the list indicating that the top-ranked predictions were likely to be true regulators (Figure 3C, 3D; Table S5). One highly-ranked candidate regulator was *Bcl11a*, a gene previously linked by human GWAS studies to increased type 2 diabetes (T2D) risk [41] [42] [43]. Of 26 loci encoding DNA-binding factors that have been linked to diabetes risk, we found that 22 (85%, *P* = 1.38×10^−8^) were expressed in the developing pancreas, and 21 of these were highly ranked by IMNA as possible regulators (Figure 3E, 3F). This provided unique evidence for roles of these diabetes risk genes in regulating pancreas development and led us to establish analytic methods to identify and prioritize transcriptional regulators for further in vivo testing (see below).

**Figure 4:**
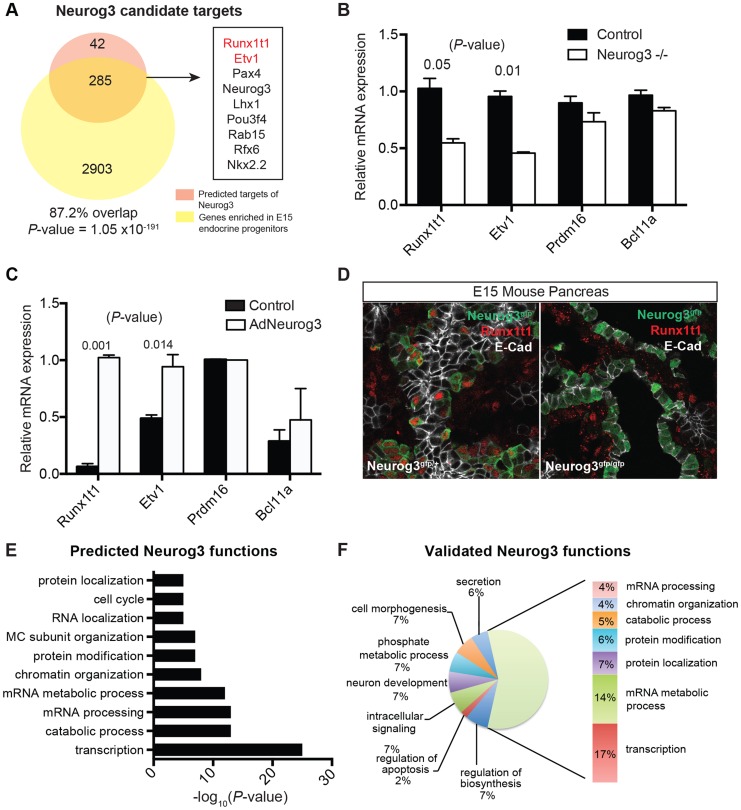
Identifying biological functions and targets of Neurog3. (A) Venn diagram displaying the number of predicted activated targets of Neurog3 using the module network algorithm of Genomica based on a cut-off value of two-fold (orange), and the number of genes that are downregulated upon the loss of Neurog3 by a two-fold difference based on expression profiling of E15 *Neurog3*-null cells (yellow). Overlap of a subset of activated Neurog3 target genes is shown to the right. Validated targets are in red. Fisher's exact test was used to calculate the *P*-value. (B) mRNA expression of a subset of nominated regulators (*Etv1*, *Prdm16*, *Runxt1t1*, and *Bcl11a*) in *Neurog3* mutant pancreata (n = 3) and control mice (n = 3) at E15. (C) Adeno-based overexpression of Neurog3 in ductal cell line (mPAC) and its effect on *Runx1t1*, *Bcl11a*, *Etv1*, and *Prdm16* expression. (n = 3,each). (D) Immunohistochemistry showing the expression of Runx1t1 (red) in a subset of Neurog3-eGFP^+^ cells in heterozygous Neurog3^eGFP/+^ (left panel). Loss of Runx1t1 (red) in the epithelium of *Neurog3*-null pancreas (right panel). No change in expression of Runx1t1 (red) in mesenchymal cells in *Neurog3*-null pancreas. Epithelial cells are labeled with E-cadherin (white). (E) Genomica-based predicted biological functions of Neurog3 based on the target genes that were positively correlated with the expression of Neurog3. (F) Biological functions of targets based on expression profiling of Neurog3^+^ endocrine progenitor cells and E15 *Neurog3*-null cells based on a 2-fold difference. (B–C) data are represented as mean +/− SEM. (D–E) functional enrichment analysis for each set of targets genes was performed through DAVID. FDR<0.05.

*Neurog3* encodes a bHLH transcription factor with essential roles in the endocrine pancreas [44], and there is intensive interest in identifying downstream targets and functions of *Neurog3* during pancreas development. Genomica module network analysis identified sets of candidate *Neurog3* target genes and further predicted these to be activated (n = 327) or repressed (n = 263) by Neurog3 (Figure 4A; Figure S6A; see Methods). Genes predicted to be induced by Neurog3 included known targets such as *Pax4*, *Rfx6*, *Nkx2.2*, *Snail2* and *Insm1*, as well as novel candidates like *Etv1* and *Runx1t1* (Figure 4A). We did not detect known regulators of pancreas development among the set of genes predicted to be repressed by Neurog3, an area not previously well-characterized [9]; thus, we prioritized analysis of the gene set predicted to be activated by Neurog3. Functional enrichment analysis for biological processes through DAVID (Figure 4E) predicted roles for these Neurog3 targets in processes including RNA biosynthesis and transcription, protein transport, localization and secretion, catabolic processes, cell cycle control and chromatin organization. These functional categories were corroborated independently by in vivo testing (see below). Thus, the module network algorithm readily identified both established and previously unrecognized *Neurog3*-dependent gene regulatory programs and target genes governing pancreatic endocrine development.

### *In vivo* validation of genes regulated by *Neurog3*

To validate predictions from IMNA, we initially focused on analyzing gene expression changes associated with the loss of Neurog3 in vivo. We purified *Neurog3*-null cells from the pancreata of E15 Neurog3^eGFP/eGFP^ mutant embryos [45] [46], an approach not previously reported [47] [48] [49]. Of the 6367 differentially expressed, 3188 were downregulated and 3179 were upregulated by the loss of Neurog3 expression (Table S6). These included both known targets such as *Pax4*, *Rfx6*, *Nkx2.2*, *Snail2* and *Insm1*, as well as predicted novel targets such as *Etv1* and *Runx1t1* (Figure 4A). Expression analysis of dissected mouse fetal pancreas by quantitative PCR confirmed that *Runx1t1* and *Etv1* mRNA were significantly decreased upon the loss of *Neurog3* (Figure 4B) and upregulated upon adenoviral misexpression of Neurog3 in pancreatic epithelial cells (Figure 4C; detailed in Methods). Moreover, immunostaining detected Runx1t1 protein in a subset of pancreatic Neurog3^+^ cells at E15, which was lost in *Neurog3*-null epithelium (Figure 4D).

**Figure 5:**
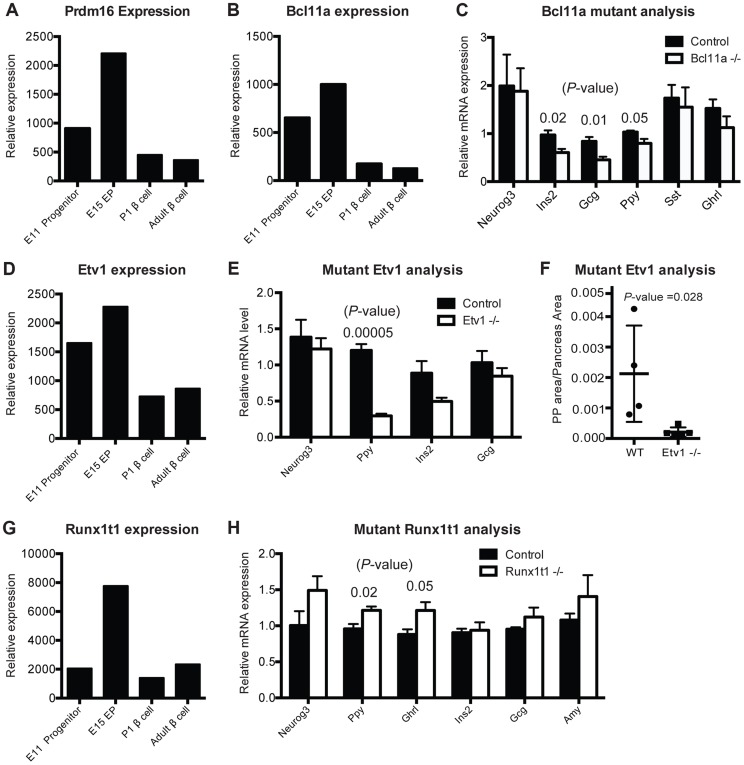
Gene-module network reveals candidate pancreas regulators. (A) Normalized expression values of Prdm16 in sorted cells. (B) Normalized expression values of Bcl11a from purified cell populations. (C) Relative mRNA expression in Bcl11a mutant mice (n = 4) and control mice (n = 4) in sorted cells enriched for endocrine cells at E15. (D) Normalized expression values for Etv1 from purified cell populations. (E) Relative mRNA expression of pancreatic markers in Etv1 mutant (n = 4) and control (n = 4) pancreata at E18. (F) Cell mass changes in PP cells in Etv1 mutant mice at birth (n = 3). (G) Normalized expression values for Runx1t1 from purified cell populations. (H) Relative gene expression in Runx1t1 mutant mice (n = 4) and controls (n = 4) at E18 from whole pancreata. In (B–G), data are represented as mean +/− SEM. In (C), (E), (H) expression levels were normalized to *beta-actin* and results are shown relative to littermate controls, (A), (B), (D), (G) represent raw values obtained from microarray analysis.

Remarkably, 87% of genes predicted by IMNA to be activated by Neurog3 (Figure 5A; *P* = 1.05×10^−191^) and 73% of targets predicted to be repressed by Neurog3 (Figure S6A; *P* = 7.02×10^−102^) were validated by our expression profiling of *Neurog3* ^+^ control and mutant *Neurog3*-null cells. Functional enrichment analysis of Neurog3-activated targets identified by expression profiling indicated roles in transcription, mRNA processing, protein transport and secretion, cell morphogenesis, catabolic processes and chromatin organization (Figure 4F). These categories matched well with those predicted by our independent module network analysis (Figure 4E). Similarly, functional enrichment analysis of biological roles of predicted Neurog3-repressed targets matched those identified by expression profiling (compare Figure S6B, S6C). In summary, this in vivo mutant mouse analysis substantially validated specific predictions made by IMNA about Neurog3 target genes and their *Neurog3*-dependent biological functions.

### Functional validation of previously unrecognized pancreas developmental regulators *in vivo*

To assess and validate candidate transcriptional regulators nominated by IMNA, we chose to assess in vivo functions of genes (1) highly ranked by IMNA (Table S5), (2) predicted to regulate target genes or functions involved in pancreas development (Figure S7), (3) without known roles in pancreas development at the initiation of these studies, and (4) with available mutant mouse alleles [50] [51] [52] [53]. These included *Prdm16*, *Etv1*, *Runx1t1*, and *Bcl11a* (Table S5). During pancreas development, mRNAs encoding each of these factors were more abundant in Neurog3^+^ endocrine progenitors than in Sox9^+^ pancreatic progenitors or beta cells (Figure 5A, 5B, 5D, 5G). This suggested possible roles for each factor in islet development. We also analyzed *Gfi*, a transcriptional regulator expressed in the fetal pancreas (Figure S8E–G), which has an established role in hematopoietic development [54] [55], but *not* nominated by IMNA as a regulator of pancreas development.

*Prdm16* encodes a transcriptional regulator and histone methyltransferase [56]. Our gene expression studies showed that *Prdm16* is highly expressed in Sox9^+^ pancreatic progenitor cells and Neurog3^+^ endocrine progenitors, then maintained at lower levels in alpha cells and beta cells (Figure 5A). IMNA predicted that *Prdm16* regulates expression of *Arx* (Figure S7A). Supporting these findings, we recently reported that homozygous null mutation of *Prdm16* leads to impaired development of pancreatic islets [17]. This included inappropriately increased expression of *Arx*, increased alpha cell and PP cell numbers (a known outcome of *Arx* misexpression; [57]), and disrupted beta cell development [17]. Thus, our work supports the prediction that *Prdm16* is required for pancreas development in vivo.

*Bcl11a* encodes a zinc-finger transcription factor involved in hematopoiesis [53] but without known roles in pancreas development. IMNA nominated Bcl11a as a candidate regulator of pancreas development, and predicted new target genes *Ins2*, *Glucagon* and *Ppy* (Figure S7B). Remarkably, we observed reduced mRNA expression of each of these genes in FACS-purified endocrine cells from homozygous null mutant *Bcl11a* ^−/−^ mice (Figure 5C). We did not find significant changes in allocation of fetal islet cell subsets in *Bcl11a* mutants (Figure S8C), and lethality at P1 precluded further phenotyping. Thus, in vivo analysis confirmed a requirement for *Bcl11a* in endocrine development.

*Etv1* (also known as Er81) encodes a transcription factor involved in neurogenesis and maturation of neural cells [50], but has no known function in pancreas development. *Etv1* mRNA levels were reduced in *Neurog3* ^eGFP/eGFP^ null pancreata (Figure 4B), indicating that *Etv1* is a direct or indirect target of Neurog3 and might have roles in islet cell development. Consistent with this possibility, we observed decreased levels of mRNAs encoding islet cell hormones, including a significant reduction of *Pancreatic polypeptide* mRNA in *Etv1* ^−/−^ mutant pancreas (Figure 5E). Likewise, morphometry of P1 null *Etv1* ^−/−^ pancreas revealed severely reduced islet PP cell mass (Figure 5F). Thus, in vivo analysis confirmed a requirement for *Etv1* in pancreatic islet development.

*Runx1t1* (also called *Eto* or *Mtg8*; [51]) encodes a transcription factor related to the *Drosophila* runt protein, and mutations in this gene have been linked to blood, lung and breast neoplasia [58] [59]. We detected Runx1t1 and Neurog3 co-expression in fetal pancreatic epithelial cells (Figure 4D). *Runx1t1* mRNA was reduced in homozygous *Neurog3* mutant pancreas (Figure 4B), and Runx1t1 protein was undetectable in homozygous null *Neurog3* ^eGFP/eGFP^ mutant cells (Figure 4D), supporting the view that Neurog3 regulates *Runx1t1* expression. IMNA analysis indicated *Runx1t1* regulates *Pancreatic polypeptide* (Figure S7D). Analysis of pancreas development in mice lacking *Runx1t1*, which expire at birth, revealed increased mRNA levels of *Pancreatic polypeptide* and *Ghrelin* expression (Figure 5H). Together with findings of significant islet cell hyperplasia in *Runx1t1* null mutants (P. Pauerstein, C.B. and S.K.K., in preparation), our analysis confirmed an essential role for *Runx1t1* in pancreas development.

*Gfi1* encodes a transcriptional regulator [60] and is expressed in fetal pancreas (Figure S8E) but was not nominated by IMNA as a regulator of pancreas development. Consistent with this prediction, we did not detect disrupted islet development or glucose regulation in mice lacking Gfi1, despite exhaustive systematic phenotyping (Figure S8F, S8G). Thus, our rigorous integration of developmental, genomic and bioinformatic approaches identified four candidate regulators of pancreas development, and mutant mouse analysis confirmed that all four were also required in vivo.

## Discussion

Elucidating the regulatory interactions underlying global transcriptional programs that control development of solid organs like the pancreas has been a challenge. Classical and recent studies have advanced our understanding of the cellular origins, genetics, morphogenesis, and cell lineage relationships in the developing pancreas, and have identified features of transient pancreatic progenitors or lineage-specific endocrine progenitors [8]. However these and other fetal pancreatic cell subsets are generated in relatively small numbers, hampering prior comprehensive genomic-scale efforts to dissect pancreas development. Here we combined several powerful approaches – including cell sorting, transgenic cell labeling, genomic-scale expression profiling, bioinformatics, and targeted mutagenesis in mice – to identify elements comprising genetic regulatory hierarchies in the developing pancreas. This effort has revealed both the complexity and structural framework of transcriptional programs underlying pancreas cell differentiation and maturation, and provides a strategy for similar studies in other solid organs.

Purification of cell subsets from defined genetic mouse strains by flow cytometry ([16] [17], this study) generated highly-reproducible gene expression profiles of a dozen pancreatic cell subsets – a degree of comprehensiveness unprecedented in prior studies. This innovation permitted deconvolution of gene expression profiles into co-expressed and co-regulated genes. We found that many gene sets were re-used in multiple lineages and stages. These findings are reminiscent of the general gene regulatory circuitry identified during hematopoiesis [10]. For example, immature alpha cell and beta cells from fetal or neonatal pancreas shared common gene sets that clustered distinctly from those in mature alpha and beta cells from adult pancreas. This feature likely reflects commonalities of mature alpha and beta cells as nutrient-responsive cells that produce, process and secrete polypeptide hormones, and corroborate similarities of gene regulation observed in adult human alpha and beta cells [61]. Global similarities of gene expression in adult alpha and beta cells shown here are also consistent with recent findings that in specific experimental settings, adult alpha cells may acquire beta cell features [62] [63] or vice-versa [64]. Identification of gene sets controlling function of mature beta cells may foster progress in producing replacement beta cells from renewable stem cell sources for diabetic patients [65]. For example, gene sets regulating calcium ion transport or responsiveness were enriched in adult beta cells, consistent with studies showing that calcium-dependent signaling pathways regulate beta cell maturation in mice and humans [31] [66]. Stimulation of calcium-responsive pathways, such as calcineurin/NFATc signaling, can enhance functional maturation of beta cells [31]. Thus, our reference data sets should prove useful for advancing efforts to produce or replace beta cells in diabetes.

Compared to the endocrine cell lineage or exocrine acinar cells, little is known about the genetic programs defining pancreatic exocrine duct cells [21]. Pearson correlation identified clustering between adult ductal cells and fetal pancreatic cells, including endocrine progenitor cells. This indicated that regulatory programs maintaining adult ductal cell gene expression and fate are unexpectedly similar to those in transient oligopotent fetal cell subsets, as suggested by unsupervised clustering with 30 mouse tissues. Thus, our study provides support for strategies focused on ‘reprogramming’ duct cells into other desired fates, including insulin-producing cells [67] [23] and could accelerate use of somatic cell reprogramming for therapeutic aims.

Human genetic studies have revealed that transcription factors have major roles in the pathogenesis of pancreatic malformation, including agenesis and diabetes mellitus [32] [68]; thus, we focused here on elucidating previously unrecognized transcriptional regulators required for pancreas development. Our general strategy was to exploit co-variance of transcription factors in gene sets and the cellular states they might regulate. Iterative use of a gene expression-based probabilistic program identified known regulators with high efficiency and predicted new regulatory functions for scores of transcription factors. IMNA identified known and novel regulators of endocrine development (see below), including a subset of transcription factors previously implicated by human GWAS in type 2 diabetes risk [41], [43]. We also noted that the frequency of detecting regulators of exocrine differentiation, like *Mist1*, was lower (Table S5). This likely reflects the lower representation of gene sets from differentiated exocrine cell types (2/12 cell subsets purified and analyzed here) compared to endocrine cell subsets. Therefore, further studies of gene regulation in subsets of purified fetal pancreatic exocrine cells could therefore likely identify additional exocrine pancreatic regulators.

To validate and assess the biological significance of predictions based on gene expression and module analysis and to control for variables introduced by our FACS-based approach, we analyzed relevant mutant mouse strains, including *Neurog3* mutants. This combined approach proved to be a powerful way to test, for example, predictions of *Neurog3* target gene expression, and to functionally validate transcriptional regulators identified by IMNA. *Bcl11a*, *Runx1t1*, *Prdm16* and *Etv1* encode transcription factors not previously linked to roles in pancreas development when these studies began. Prior studies had revealed crucial roles for *Bcl11a* in regulating blood development [69] [53] and diseases [70] [71], and for *Runx1t1* in midgut development [51] and neoplasias of blood, lung and breast [58] [59]. *Prdm16* has well characterized functions in adipogenesis [72] [73], leukemia pathogenesis [74], and neuronal stem cells maintenance [75]. *Etv1/Er81* is an established regulator of fetal neuronal development [50] whose mis-expression leads to cancer pathogenesis in diverse tissues [76]. Independent genetic screens in our group, concurrent with studies here, identified roles for *Prdm16* in regulating allocation of pancreatic islet cells in development [17].

Strikingly, after unbiased selection of these four candidate regulators, analysis of mouse strains harboring targeted mutations in *Etv1*, *Prdm16*, *Bcl11a* and *Runx1t1* here or in recent studies from our group [17] revealed defects of pancreas development in all of the mutants. Though expressed in islet development, *Gfi1*, an established regulator of myeloid and enteric development [54] [77] [78], did not meet criteria of a regulator through our bioinformatic analysis. Accordingly, intensive investigation revealed no detectable phenotypes in pancreas development or glucose control in mice lacking *Gfi1*. Thus, our integrated approach accurately predicted essential regulators of islet development, demonstrating the robustness of our cell purification and gene expression profiling. This level of functional validation with mutant mouse phenotyping is, to our knowledge, unprecedented for integrative genomic approaches to pancreas development. Clearly, additional in-depth phenotypic studies, including in mice permitting conditional or pancreas-specific gene targeting, could prove valuable for understanding the molecular roles of these factors in pancreas development. Improved methods to isolate, purify and analyze cognate human pancreatic cell subsets [79] should enable an analogous integrative approach to identify factors regulating human pancreatic development.

Endoderm-derived epithelial cells and their progeny accomplish the vital physiological functions of the adult pancreas, and in other gastrointestinal organs. Thus, in these initial investigations we focused on deciphering the transcriptional hierarchies underlying epithelial cell development in the pancreas. However, prior studies have revealed that non-epithelial cells, including vascular endothelium, neuronal cells, and mesenchyme-derived signals control basic aspects of pancreas development [7] [80]. Thus, a complete deconstruction of pancreas development will require assessments, akin to those described here, of gene expression data from additional important cell subsets. Likewise additional data from epigenetic, genome-scale ChIP-Seq, proteomics and enhancer analyses [81] need to be integrated into the regulatory frameworks described here. The coordinated developmental, cellular, molecular and computational approaches described here should provide a paradigm for identifying genes and circuitry underlying development and postnatal maturation in other visceral organs, as well as assessment of regenerated pancreatic cell types.

## Methods

### Animals

All animal studies were approved by Stanford University and performed in accordance with Stanford University Animal Care and Use guidelines. Discomfort of animals was limited to that which was unavoidable in the conduct of scientifically valuable research. Analgesic, anesthetic, and tranquilizing drugs used where indicated and where appropriate to minimize discomfort and pain.

Mice harboring the *Sox9*-eGFP BAC transgene were obtained from Mutant Mouse Regional Resource Center, University of California at Davis [82]. Because of eGFP perdurance, *Sox9*-eGFP^+^ cells in *Sox9*-eGFP mice contain a mixture of Sox9^+^ and Sox9^neg^ progeny. However, the percent of Sox9^neg^ cells is low [17]. *Neurog3* ^eGFP^ transgenic mice were a kind gift from Drs. Guoqiang Gu and Douglas Melton [13]. Neurog3 knock-in reporter mice were a kind gift from Dr. Klaus Kaestner [45] [46] and provided by Dr. O. Cleaver. Mouse Insulin Promoter (MIP)-GFP mice were a gift from Dr. M. Hara (University of Chicago, Chicago, IL; [83]) and maintained in a CD-1 background. Glucagon-Venus mice were a gift from Dr. Fiona Gribble [84]. *Runx1t1* mutant mice were rederived from MRC Harwell (Stock number FESA:000373). *Etv1* mutant mice were a gift from Dr. Thomas Jessell and provided by Dr. Julia Kaltschmidt (Memorial Sloan Kettering Institute). *Bcl11a* mutant mice were derived from Bcl11a floxed mice [53] by crossing with a Cre deleter strain (CMV-Cre), obtained from Jackson Laboratories (Stock number 006054). *Prdm16* mutant mice were obtained from Jackson Laboratories (Stock number 013100). *Gfi1* mutant mice are described in [54]. Genotyping follows published methods. Mice were mated overnight and checked for plugs. Noon on the day of vaginal plug appearance was counted as embryonic day 0.5 (E0.5).

### Cell sorting strategy and flow cytometry

Pancreata were obtained at E11, E15, E17, postnatal day (P) 1, P15, and 8–12 weeks of age. To obtain E11 Sox9^+^ pancreatic progenitors, we dissected the dorsal pancreas from *Sox9*-eGFP reporter embryos and dissociated with TrypLE Express (Invitrogen, Carlsbad, CA) at 37°C for 5 min and triturated. TrypLE was neutralized with 10% (v/v) FBS in PBS [17]. Approximately 200 pancreata were dissected to obtain four replicates of Sox9^+^ pancreatic progenitors. E15 pancreatic progenitors, Neurog3^+^ endocrine progenitors, and acinar cells were collected using a combination of cell surface markers and transgenic cell labeling. This approach was used to collect hormone^neg^ Neurog3^+^ endocrine progenitors because GFP perdurance labels their hormone^+^ descendants. Briefly, the Neurog3^eGFP^ transgenic pancreata were dissected and visually assessed for GFP expression. GFP^+^ pancreata were pooled and dissociated with 0.05% Trypsin EDTA at 37°C for 8 min and triturated (GFP^neg^ pancreata were collected for FACS gating purposes). Trypsin EDTA was neutralized with 10%(v/v) FBS in 10 mM EGTA, PBS [16] and the cells were treated for 15 min in a blocking solution composed of FACS buffer (2% fetal bovine serum in PBS, 10 mM EGTA was supplemented for experiments of E15 pancreas) containing 300 ng/ml rat IgG (Jackson ImmunoResearch, West Grove, PA). We used the following primary antibodies: biotin anti-CD133 (13A4, 1∶100; eBioscience, San Diego, CA), Pacific Blue anti-CD24 (M1/69, 1∶100; BioLegend, San Diego, CA), and PE anti-CD49f (GoH3, 1∶50; R&D, Minneapolis, MN). Streptavidin-APC (1∶200; eBioscience) was used to visualize biotinylated antibodies. Gating was performed in accordance to Sugiyama [16]. The antibody combinations are shown in Figure 1B. E15 Neurog3-null cells were collected similarly from GFP^+^ Neurog3^eGFP/eGFP^ knock-in embryos. A total of 405 pancreata were dissected to collect each replicate of Neurog3^+^ endocrine progenitors. Each replicate comprises ∼15,000 cells.

Beta cells and alpha cells were obtained from MIP-GFP and Glucagon-Venus reporter mice, respectively. Beta cells were collected from E15, E17, postnatal day (P) 1, P15, and 8–12 week-old male mice, while alpha cells were collected at P1 and 8–12 week-old male mice. E15 and E17 MIP-GFP^+^ pancreata were dissociated with 0.05% Trypsin EDTA for 8–10 min at 37°C and triturated from 300 pancreata and 240 pancreata, respectively. P1 MIP-GFP^+^ and P1 Glucagon-Venus^+^ pancreas were dissociated with 1 mg/ml collagenase (Sigma-Aldrich; C01030) for 8 minutes, followed by mixing, spinning and further dissociation with 1 mg/ml dispase for 8 minutes at 37°C from approximately 230 pancreata. P15 and adult pancreata were dissociated by standard intraductal ligation and digestion with 1 mg/ml collagenase [31]. Each replicate consisted of at least 3 male mice. Beta cells from the stages E15, E17 and P1 are termed ‘fetal’ beta cells, while beta cells from P15 and adult mice are ‘postnatal’ beta cells based on their ability to couple glucose detection with insulin secretion [85]. Adult duct cells were collected using APC anti-CD133 (1∶100; BioLegend, San Diego, CA). To exclude blood cell contamination from our sorts we used cell-surface markers Ter119 and CD45 (1∶100; Biosciences), which label erythroid cells and leukocytes, respectively. Live-dead cell exclusion was performed with 10 µg/mL Propidium Iodide (PI; Sigma) or 10 µg/mL Aqua (L34957; Invitrogen).

Cell sorting was performed in FACS Aria I and II machines fitted with a 100 uM nozzle using DIVA software (BD Biosciences, San Jose, CA). FACS data were analyzed by using FlowJo software (Tree Star, San Carlos, CA). Cells were collected in 10% fetal bovine serum in PBS and processed for RNA collection (for acinar cells, 2% fetal bovine serum in PBS supplemented with 10 mM EGTA). Cell death did not exceed 30% per sample. All cell types were collected in at least triplicate from a minimum of 15,000 cells per sample.

### Molecular biology

Total mRNA was isolated using the Arcturus PicoPure kit (Applied Biosystems) for all microarray samples. For quantitative PCR analysis, whole pancreas mRNA at E18 or P1 was collected by homogenizing each pancreas in 1.5 ml of RLT buffer and RNA was extracted using the Qiagen RNAeasy Micro kit (Qiagen). cDNA synthesis was performed with Ambion Retroscript kit. Quantitative PCR studies were performed using an ABI7500 system, Applied Biosystems (Foster City, CA). Replicates were processed independently, and each cDNA was tested in duplicate. Expression level was normalized to *beta-actin*. Information about primer and probe sets is available upon request.

### Microarray data preprocessing, normalization and clustering

RNA quality was accessed with Agilent's BioAnalyzer (Stanford PAN facility). 50 ng to 100 ng of mRNA with a RNA integrity number (RIN) score >9 were amplified with NuGen Ovation Kit V2 (NuGEN) and fragmented and labeled with the Biotin and fragmentation labeling kit (NuGEN) following manufacturer's protocol. Hybridization and image analysis processing was done in accordance to the Stanford PAN facility. The Affymetrix Mouse 430 2.0 GeneChip was used.

For gene expression analysis, arrays were RMA normalized using justRMA package in R. After normalization, probes with raw expression value of 100 in all arrays were filtered out—leaving a total of 23,093 probes. For each expressed probe, expression values were log2-transformed, and mean-centered across all the conditions before pair-wise Pearson correlation was performed. Unsupervised hierarchical clustering and array clustering of pair-wise Pearson correlation was performed using Cluster 3.0 [86]. This dendrogram was overlaid with the Pearson correlation plot (Figure 1C).

Unsupervised hierarchical clustering analysis with Microarray data from over 30 adult mouse tissues was downloaded from NIH GEO, with accession number GSE1133 [19]. The data discussed in this publication have been deposited in NCBI's Gene Expression Omnibus [87] and are accessible through GEO Series accession number GSE54374 (http://www.ncbi.nlm.nih.gov/geo/query/acc.cgi?acc=GSE54374).

### Module mapping

The module mapping function of Genomica [34] was executed to identify gene sets that are enriched or depleted in each sample. Here, we entered gene ontology terms belonging to biological processes (BP-GO terms) from mouse and the mean-centered normalized expression values for all the arrays (23,093 probes). We used the default settings of the program: *P*-value of 0.05, FDR correction of 0.05, and the hierarchical agglomerative (correction centered) clustering method. Probes with an expression level > = 1 (on a log2 scale) were considered upregulated, while probes with an expression level < = −1 (on a log2 scale) were considered downregulated. We displayed the average expression of gene hits from each enriched gene set (based on a log2 scale of mean-centered values). GO gene sets of biological functions were downloaded from website DAVID and imported into the Genomica Software [20] [34].

### Gene signatures and pair-wise comparisons

Gene signatures for each cell type were calculated using Student t-test comparing signals in the arrays of a particular cell-type versus the rest of the arrays. The genes selected as ‘signature genes’ met four parameters: *P*-value< = 0.001, FDR< = 0.05, log2 fold change > = 1, and standard deviation < = 0.5 of arrays in the same cell type. FDR correction was estimated using the p.adjust package in R. Negative gene signatures were selected using a log2 fold change < = −1, *P*-value< = 0.001, FDR< = 0.05, and standard deviation < = 0.5 of arrays in the same cell type. Similar methods were applied for transcription factor signatures. For each gene signature, we performed functional enrichment analysis of Gene Ontology terms related to biological processes (GOTERM_BP_FAT) through DAVID. BP-GO terms were considered significant if FDR>0.05. DAVID default settings were used.

To obtain differentially expressed genes between two cell types we used the Student t-test where the obtained *P*-values were adjusted for multiple testing using the p.adjust function of R with Benjamini-Hochberg method (adjusted *P*<0.05). The log2 fold change difference between each representative cell type was calculated by averaging the transformed probe signals in the arrays of the first cell type and subtract that from the second cell type. Fetal endocrine cells included E15 beta cells, E17 beta cells, P1 beta cells, and P1 alpha cells. Postnatal endocrine cells include P15 beta cells, Adult beta cells, and Adult alpha cells.

### IMNA expression-based prediction of regulators, their target genes and functions

We identified candidate regulators and their regulation programs using the Module Networks algorithm in the Genomica software [10] [34]. Genomica detects modules of co-expressed genes (gene sets) and their shared regulatory programs. A regulation program is a small set of genes whose expression is predictive of the expression level of the module genes using a decision (regression) tree structure. Given the expression values of a pool of candidate regulator genes, a set of modules and their associated regulation programs are automatically inferred by an iterative procedure. This procedure searches for the best gene partition into modules and for the regulation program of each module while optimizing a target function. The target function is the Bayesian score derived from the posterior probability of the model (see [34] for a detailed description of the algorithm). The program requires two input lists: 1) list of potential regulators as determined by the user and 2) normalized mean-centered expression data for the samples of interest. We compiled a list of 1642 mouse transcription factors (TF) or TF components from various sources [36], [37] [38]. Our list of input ‘regulators’ was a filtered list of TFs and TF components that were expressed in our arrays (total of 3310 expressed probes based on our list of 1642 candidate TFs). Our list of input ‘expression values’ consisted of the normalized and mean-centered values for all 12 cell populations (described above; 23,093 probes). We applied the default settings and set the maximum tree depth to 5.

We optimized the maximum number of modules by counting the number of known regulators predicted. The list of known pancreatic regulators (82 total) was compiled from [9] and literature searches on all predicted regulators in pubmed. Genomica identifies the sets of candidate regulators that are most predictive of the expression pattern for each module (co-expressed genes). We tested the quality of its predictions by setting: 25, 50, 75, and 100 modules. After a single run, 100 modules identified 25% of known regulators of pancreas development. Because this number was low and because single runs were inherently unstable, we reasoned that multiple iterations may yield better predictions. We tested the optimal number of iterations counting the number of known regulators after 1, 20, 40, 60, 80, and 100 iterations for each module number. Reproducibility was determined by ranking the frequency that each candidate regulator is identified after each run and by performing Gene set enrichment analysis (GSEA; [40]; Figure S5) of true positives (known regulators). We determined that 100 iterations of 75 modules was the best setting; it identified 99% of known pancreatic regulators and provided the best GSEA enrichment score. Increasing the number of iterations to 110 or 120 predicted the same number of known pancreatic regulators but worsened the enrichment score. To obtain the GSEA enrichment score of diabetes risk factor genes, we compiled a total of 72 risk factor genes from [42] and [88]. Then we performed GSEA on factors that were transcription factors or DNA-binding proteins (26/72 genes) and were expressed in the pancreas (22/26 TFs genes). Using our list of 22 risk factor genes we obtained the enrichment score relative to the ranked list from our IMNA approach as described above.

The predicted targets and functions for a subset of candidate regulators were determined by extracting the modules that each regulator was predicted to regulate. All the modules that were positively regulated were grouped (comprising of a minimum of 5 modules per regulator). This was similarly done for modules that a regulator was predicted to repress. We obtained the predicted biological functions for each regulator by performing functional enrichment analysis on each list of genes through DAVID. BP-GO terms were considered significant if FDR>0.2. DAVID default settings were used.

To validate the predicted targets and functions of Neurog3, we performed Student's t-test to obtain differentially expressed genes between E15 Neurog3^+^ endocrine progenitors and E15 Neuorg3-null cells. We obtained ∼8000 probes that were differentially expressed with a 2-fold difference (4604 probes enriched in E15 endocrine progenitors and 4217 probes enriched in Neurog3-null cells). We compared this list to targets predicted by Genomica. This analysis yielded an 85% overlap in probes (303/358; *P* = 2.01×10^−168^; Figure 4A) or 87.2% overlap in genes (285/327; P = 1.05×10^−191^) when we filter out probes in each predicted module that have an expression value = <1 (based on a log2 scale). The same approach was used to predict repressed targets of Neurog3. When we filter probes with an expression value >−1, 67% of probes overlap (220/328, P-value = 2.60×10−85; Figure S6A) or 73% genes overlap (192/263; *P* = 7.02×10^−102^). Fisher's exact test was used to determine statistical significance with *P*<0.05 against the total number of probes or genes. Next, we performed functional enrichment analysis on each list of Genomica predicted targets through DAVID using default settings and compared these results to targets obtained by gene expression profiling of Neurog3-null cells (FDR<0.05).

The analysis was integrated through GenomeSpace (http://www.genomespace.org/). Venn diagrams were obtained with the Venny program [89] (http://bioinfogp.cnb.csic.es/tools/venny/index.html). Fisher's exact analysis was performed using the following website: http://www.langsrud.com/fisher.htm

### Statistical analyses

Each variable was analyzed using the two-tailed Student's t test. For all analyses, a *P* value of less than 0.05 was considered significant. Results are given as mean +/− SEM.

### Over-expression of Neurog3

A mouse duct cell line (mPAC) was infected with adenoviruses expressing the mouse Neurog3 gene and the red fluorescent protein (RFP) from separate CMV promoters. The control sample included mPAC cells that were infected with Adenoviruses expressing RFP. We verified that RFP did not have an effect on the expression of Neurog3 downstream genes in non-treated mPAC cells. Cells were cultured with the virus for 1 day and then media was changed. Cells were harvested after 3–4 days. Each experimental condition was performed in triplicate.

### Immunohistology

For measurement of endocrine-cell mass, a minimum of 12 pancreas sections spanning the entire pancreas were assessed for at least 3 different mice per genotype. The total cross-sectional area of hormone^+^ cells was summed and normalized to total pancreatic area using Image-Pro Plus analysis software (Media Cybernetics). Statistical analysis was performed using a two-tailed Student's t-test. For staining Runx1t1 and Etv1-LacZ expression, E15 and 2-month old mouse pancreata were dissected and fixed with 4% paraformaldehyde overnight at 4°C, and cryo-embedded. Sections were permeabilized with 1% Triton-X-100 for 1 hr before blocking with 2%BSA, 1% DMSO in PBS. We used the following primary antibodies: Goat anti-Runx1t1 (1∶200, Santa Cruz, C-20), Rabbit anti-LacZ (1∶500, Invitrogen), and Rat anti-E-cadherin (1∶400, Invitrogen). Secondary antibodies were from Jackson ImmunoResearch and Molecular Probes. Samples were mounted with Vectashield containing DAPI (Vector Laboratories). Microscopic images were obtained using a Leica SP2 AOBS confocal laser-scanning microscope.

## Supporting Information

Figure S1 Heat map of mRNA expression of a subset of known pancreatic markers. (A) Heat map of genes that are representative of each major cell type collected. High relative expression is shown in red and low relative expression in blue based on a log2 scale. (B) Insulin and Glucagon mRNA-expression analysis of sorted beta cells from adult mice. (C) Insulin and Glucagon mRNA-expression of sorted alpha cells from adult mice.
(PDF)

Figure S2 Hierarchical clustering of pancreatic cells with 30 adult mouse tissues. The data was normalized and clustered. The cells in this study are in red and those from [19] are in black.
(PDF)

Figure S3 Gene signatures across 12 sorted cell types. (A) Genes that are enriched in each cell type were termed ‘positive gene-signatures’ based on four parameters (*P*-value< = 0.001, FDR< = 0.05, log2 fold change > = 1, and standard deviation < = 0.5 of arrays in the same cell type). Range of expression values (−5.015, 4.84). More than 75% of probes with positive values lie within the range of +2.1 to −2.1 based on a log2 scale. (B) Genes that are repressed in each cell type were termed ‘negative gene-signatures’ based on parameters (*P*-value< = 0.001, FDR< = 0.05, log2 fold change > = −1, and standard deviation < = 0.5 of arrays in the same cell type). Range of values (−6.55, 3.98).>75% of probes with negative values lie within the range of −2.1 to +2.1 based on a log2 scale. (A–B) E15, E17, and P1 beta cell samples were grouped to obtain the gene signature of fetal beta cells and P15 and 8–12 week beta cells were grouped to obtain the gene signature of postnatal beta cells. SPP (Sox9^+^ Pancreatic Progenitor), EP (Endocrine Progenitor). Scale bar based on a log2 scale. The number of genes corresponding to each gene signature are shown below each heatmap. Corresponding values for each figure are shown in Table S2.
(PDF)

Figure S4 Pair-wise comparisons between endocrine cells. Volcano plots representing the distribution of probes against their *P*-value and FDR cut off of <0.05 (horizontal red line). Horizontal red lines represent an expression cut off with a log2 value of −1 and +1. X-axis represents the log2 fold change between each pair of conditions, while the Y-axis represents the −log10 value of the P-value. The annotated probes with the highest fold change difference are noted in each graph. A full list of differentially expressed genes for each condition is shown in Table S4. Color scheme of cell types as shown in Figure 1B, i.e. blue (beta cells), green (alpha cells).
(PDF)

Figure S5 GSEA of various module and iterations parameters used in IMNA. Gene set enrichment analysis displaying the enrichment score and distribution of known regulators of pancreas development based on their frequency. We show 25 modules at 100 iterations, 50 modules at 100 iterations, 75 modules at 120 iterations, and 100 modules at 100 iterations. The enrichment score for these parameters was worse than the enrichment score for 75 modules at 100 iterations. All statistical tests had a *P*-value and FDR value of <0.05.
(PDF)

Figure S6 Validation of repressed *Neurog3* functions and targets. (A) Venn diagram showing genes that were upregulated in E15 *Neurog3*-null cells (yellow) and predicted repressed targets of *Neurog3* based on module network analysis in Genomica (orange). Fisher's exact test was used to calculate the *P*-value. (B). Functional enrichment analysis of biological functions of predicted repressed targets of Neurog3 based on the module network analysis algorithm. (C) Functional gene set analysis of genes that were enriched in *Neurog3*-null cells vs. E15 Neurog3^+^ endocrine progenitors (by 2-fold) was performed using DAVID (FDR<0.05), similar biological terms were grouped.
(PDF)

Figure S7 Predicted targets and GO terms of a subset of regulators. (A–D) Predicted biological functions of *Bcl11a*, *Runx1t1*, *Etv1*, and *Prdm16* as determined by DAVID analysis of Genomica predicted targets for positively-correlated genes. FDR<0.2. X-axis shows the −log (p-value) of each biological function as calculated in DAVID. A sample of the predicted targets is shown to the right. Validated targets are shown in red.
(PDF)

Figure S8 Phenotypic mutant analysis of nominated regulators. (A) Expression of *Etv1* expression in adult mouse pancreas using the Etv1^LacZ^ knock-in reporter mouse with Glucagon staining (red). (B) Immunostaining showing that Runx1t1 (green) is expressed in a subset of islets sells as determined by overlap with islet marker Chromogranin A (ChgA, green), epithelial cells are shown in white in E15 fetal pancreas. (C) Morphometric analysis comparing the insulin^+^ cell area and glucagon^+^ cell area in *Bcl11a* mutant mice compared to littermate controls (n = 3 each) at birth (P1). (D) Morphometric analysis of pancreatic polypeptide^+^ (PP), insulin^+^ (Ins), and glucagon^+^ (Gcg), and somatostatin^+^ (Sst) cell area in Etv1 mutant mice on embryonic day 18 (n = 5, each). In (C) and (D) there were no statistically significant changes in each comparison. (E) mRNA expression of Gfi1 from E15 pancreatic progenitors, E15 endocrine progenitors, E15 endocrine cells, E15 acinar cells, and E15 *Neurog3*-null cells. (F) Fasting glucose tolerance between 8–12 week old *Gfi1* mutant mice and control littermates (n = 3, each). (G) mRNA expression analysis comparing a set of pancreatic markers between *Gfi1* mutant whole pancreas and control mice at P1 (n = 2, mean +/− SEM).
(PDF)

Table S1 Pair-wise comparison of alpha and beta cells by developmental stage. Fetal beta cells represent E15, E17, P1 beta cells while postnatal beta cells represent P15 and 8–12 week beta cells. Percentage of affymetrix probes that are differentially expressed based on a total probe number of 45101.
(XLS)

Table S2 Gene signatures of pancreatic cells types. Tab1: Positive signature. Tab2: Negative signature. Fetal beta cells (E15, E17 and P1 beta cells). Postnatal beta cells (P15 and 8–12 week beta cells). Parameters used to obtain gene signatures are described in methods. Corresponding Figure S3A–B.
(XLS)

Table S3 Pair-wise comparisons of cells of progenitors. Tab1: E11 SPP vs. E15 SPP. Tab 2: E11 SPP vs. E15 acinar cells. Tab 3: E11 SPP vs. adult duct cells. Tab 4: E11 SPP vs. E15 EP. Tab 5: E15 SPP vs. adult duct cells. Expression values represent Log_2_ normalized values. Only values that are differentially expressed by a 2-fold change and have an adjusted P-value of <0.05 (based on a multiple hypothesis correction) are shown. SPP (Sox9^+^ pancreatic progenitor), EP (endocrine progenitor).
(XLS)

Table S4 Pair-wise comparisons of cells of endocrine cells. Tab1: Postnatal (P15 and adult beta cells) vs. fetal (E15, E17, P1) beta cells. Tab 2: Fetal beta vs. P1 alpha cells. Tab 3: P1 alpha vs adult alpha cells. Tab 4: Postnatal beta cells vs. adult alpha cells. Tab 5: Fetal (E15, E17, and P1 beta cells and P1 alpha cells) vs. postnatal endocrine cells (P15 and adult beta cells and adult alpha cells). Tab 5: E15 EP vs E15 beta cells. Expression values represent Log_2_ normalized values. Only values that are differentially expressed by a 2-fold change and have an adjusted P-value of <0.05 (based on a multiple hypothesis correction) are shown. Corresponding volcano plots are displayed in Figure S4. EP (endocrine progenitor).
(XLS)

Table S5 Predicted regulators of pancreas development by IMNA. The frequency ratio was calculated based on the number of times each regulator appears after each iterative run. A total of 100 iterative runs were performed each comprising of 75 gene-network modules. Analysis was executed using the module network algorithm of Genomica.
(XLS)

Table S6 Pair-wise comparison of Neurog3-wt vs. Neurog3 null cells. Differentially expressed genes were obtained by comparing the expression values of E15 Neurog3^+^ endocrine progenitor (E15 EP) cells to sorted E15 Neurog3-null cells. Expression values represent Log_2_ normalized values. Only values that are differentially expressed by a 2-fold change and have an adjusted P-value of <0.05 (based on a multiple hypothesis correction) are shown.
(XLS)
