# Supplementary figures and images for: An Integrated Cell Purification and Genomics Strategy Reveals Multiple Regulators of Pancreas Development
Source: PLoS Genet. 2014 Oct 16;10(10):e1004645. doi: 10.1371/journal.pgen.1004645 (PMC4199491; doi:10.1371/journal.pgen.1004645)

Figure S1

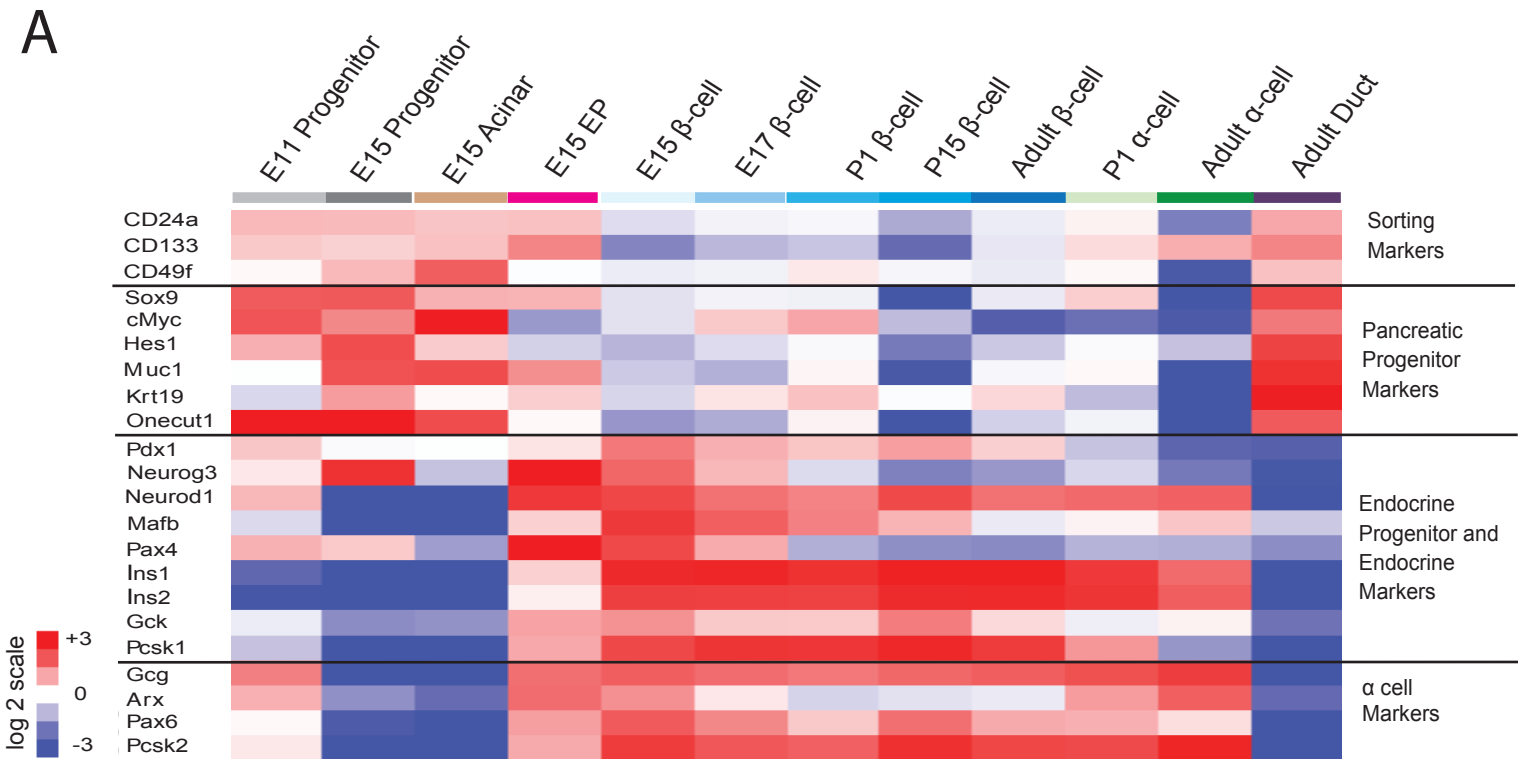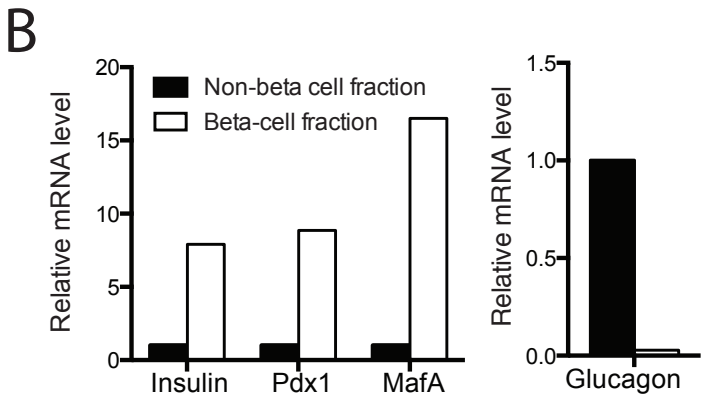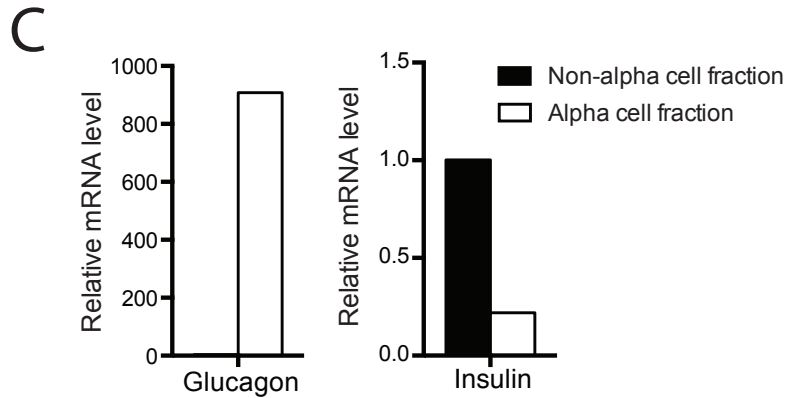

Supplement: Figure S1 — Heat map of mRNA expression of a subset of known pancreatic markers. (A) Heat map of genes that are representative of each major cell type collected. High relative expression is shown in red and low relative expression in blue based on a log2 scale. (B) Insulin and Glucagon mRNA-expression analysis of sorted beta cells from adult mice. (C) Insulin and Glucagon mRNA-expression of sorted alpha cells from adult mice. (PDF) [file pgen.1004645.s001.pdf]

Figure S2

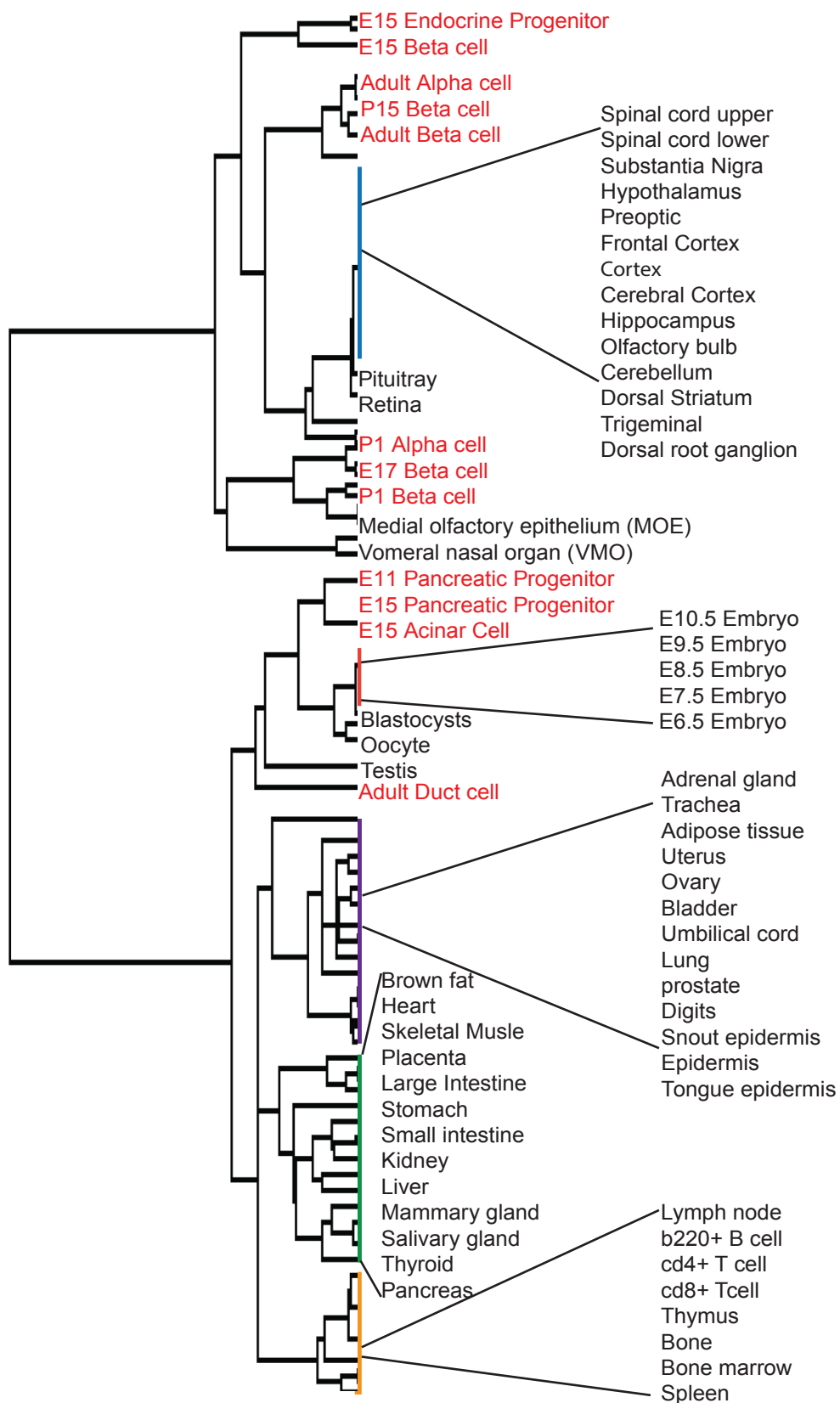

Supplement: Figure S2 — Hierarchical clustering of pancreatic cells with 30 adult mouse tissues. The data was normalized and clustered. The cells in this study are in red and those from [19] are in black. (PDF) [file pgen.1004645.s002.pdf]

Figure S3

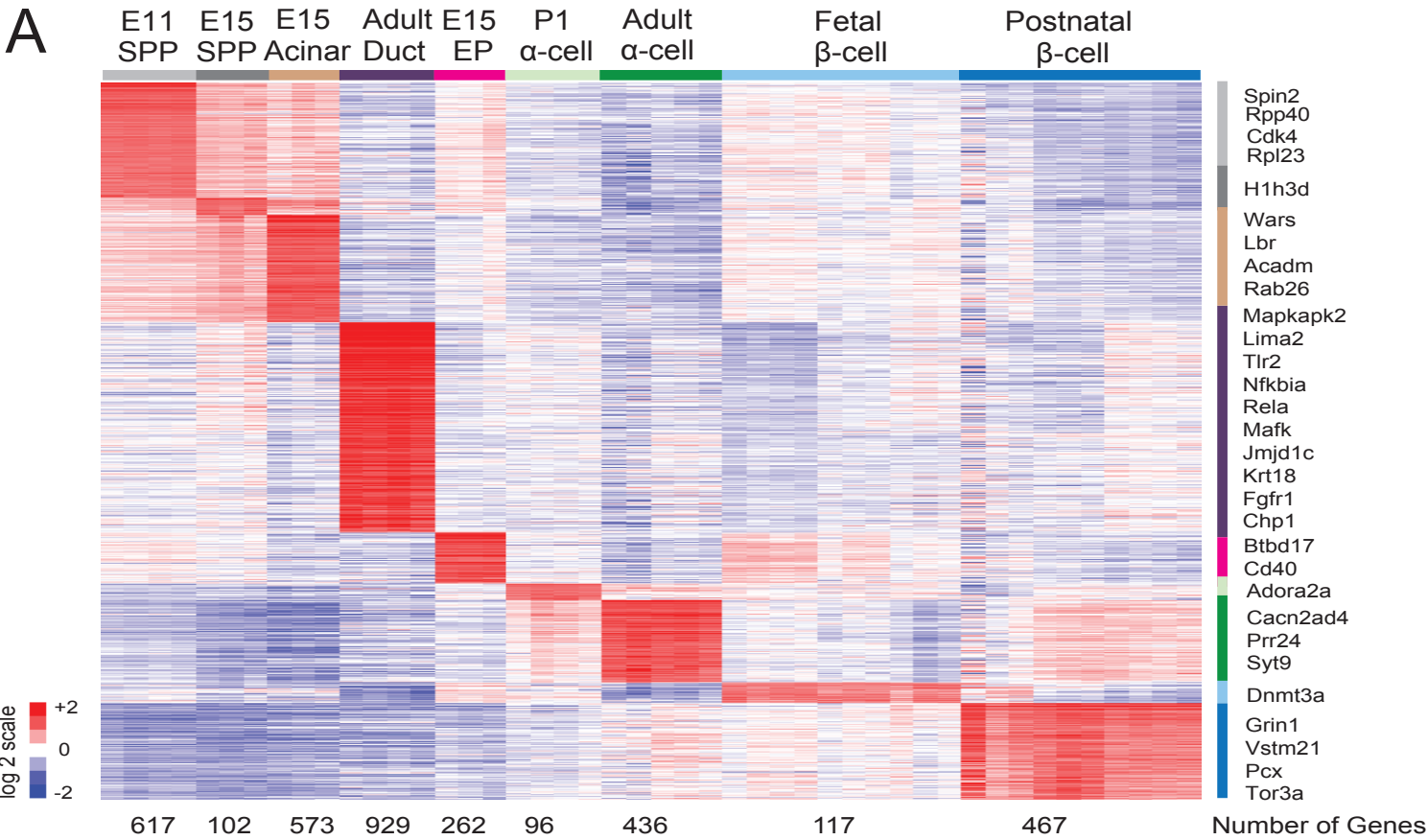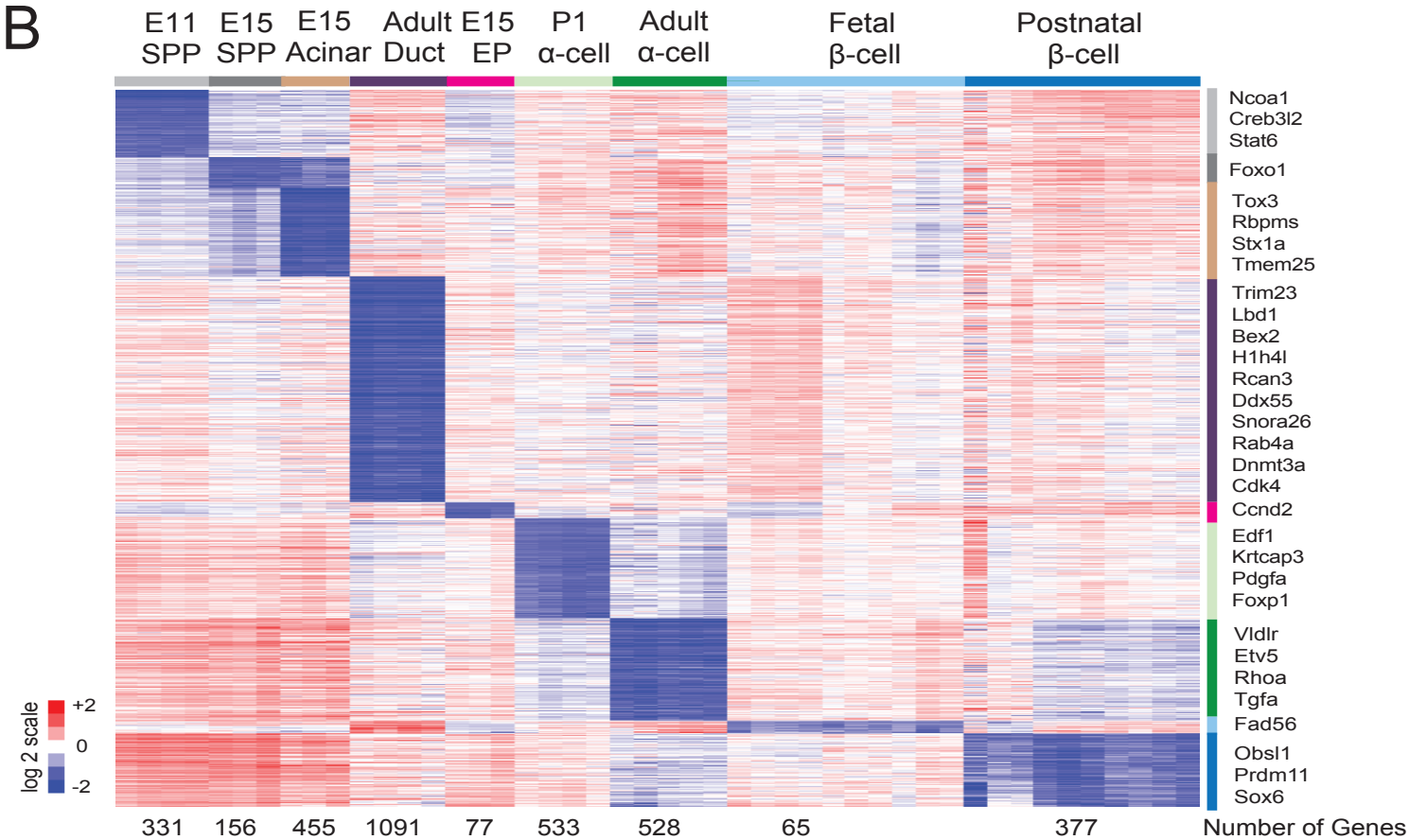

Supplement: Figure S3 — Gene signatures across 12 sorted cell types. (A) Genes that are enriched in each cell type were termed ‘positive gene-signatures’ based on four parameters (P-value< = 0.001, FDR< = 0.05, log2 fold change > = 1, and standard deviation < = 0.5 of arrays in the same cell type). Range of expression values (−5.015, 4.84). More than 75% of probes with positive values lie within the range of +2.1 to −2.1 based on a log2 scale. (B) Genes that are repressed in each cell type were termed ‘negative gene-signatures’ based on parameters (P-value< = 0.001, FDR< = 0.05, log2 fold change > = −1, and standard deviation < = 0.5 of arrays in the same cell type). Range of values (−6.55, 3.98).>75% of probes with negative values lie within the range of −2.1 to +2.1 based on a log2 scale. (A–B) E15, E17, and P1 beta cell samples were grouped to obtain the gene signature of fetal beta cells and P15 and 8–12 week beta cells were grouped to obtain the gene signature of postnatal beta cells. SPP (Sox9+ Pancreatic Progenitor), EP (Endocrine Progenitor). Scale bar based on a log2 scale. The number of genes corresponding to each gene signature are shown below each heatmap. Corresponding values for each figure are shown in Table S2. (PDF) [file pgen.1004645.s003.pdf]

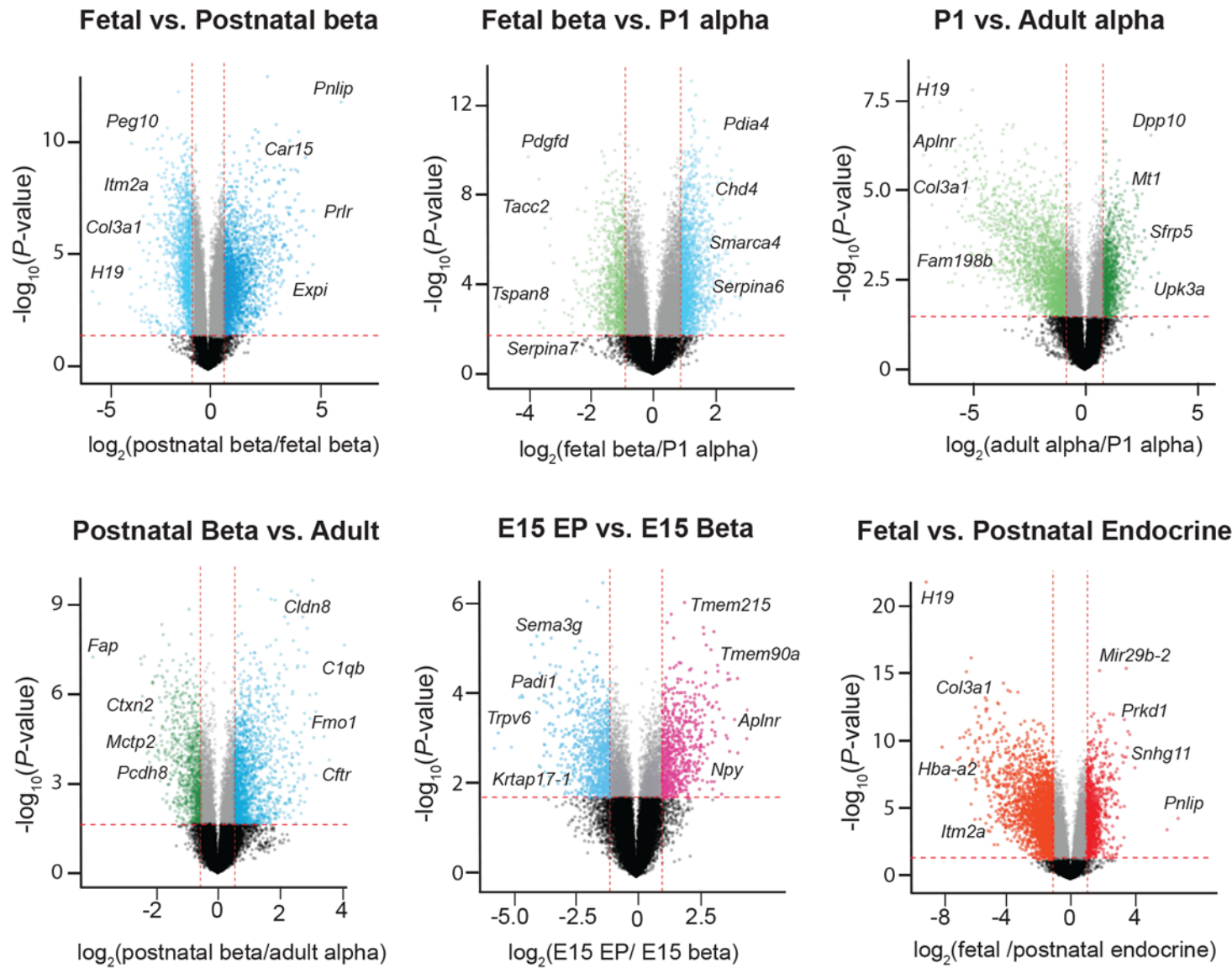

Supplement: Figure S4 — Pair-wise comparisons between endocrine cells. Volcano plots representing the distribution of probes against their P-value and FDR cut off of <0.05 (horizontal red line). Horizontal red lines represent an expression cut off with a log2 value of −1 and +1. X-axis represents the log2 fold change between each pair of conditions, while the Y-axis represents the −log10 value of the P-value. The annotated probes with the highest fold change difference are noted in each graph. A full list of differentially expressed genes for each condition is shown in Table S4. Color scheme of cell types as shown in Figure 1B, i.e. blue (beta cells), green (alpha cells). (PDF) [file pgen.1004645.s004.pdf]

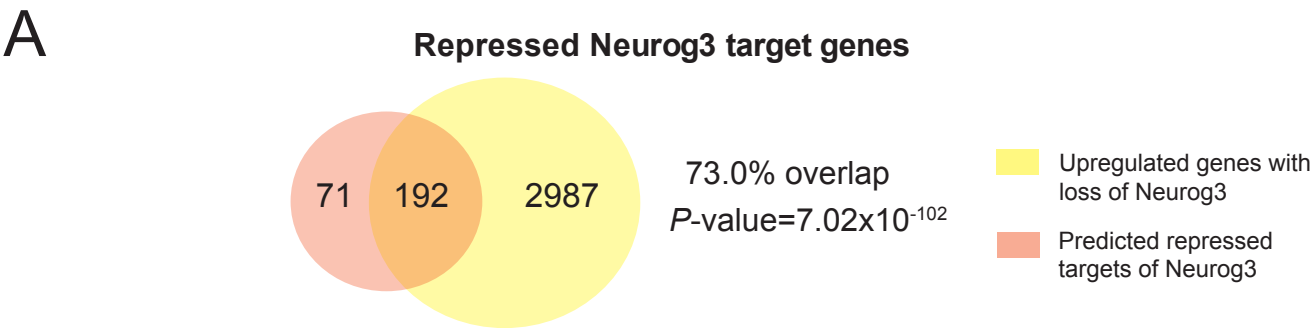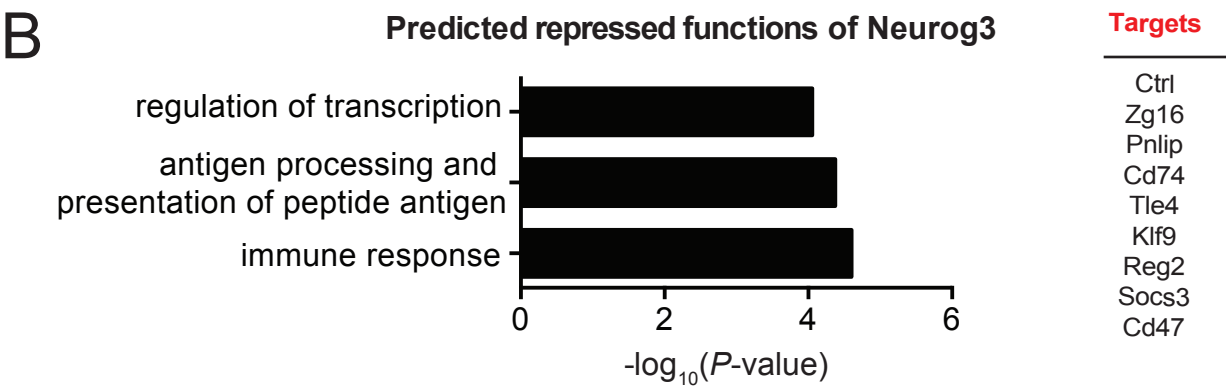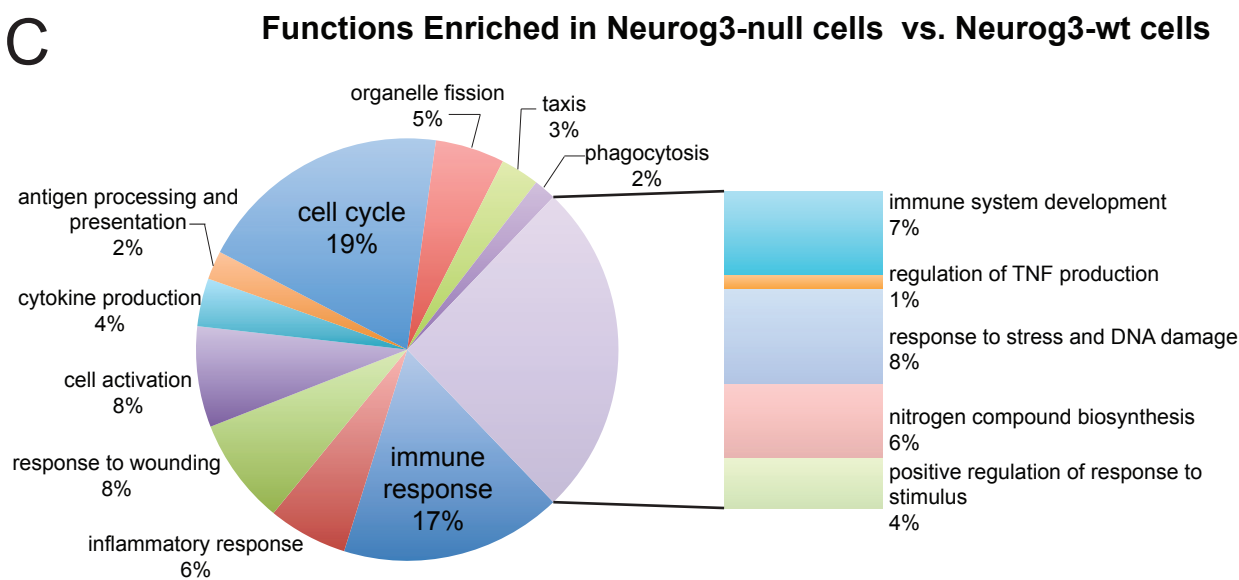

Supplement: Figure S6 — Validation of repressed Neurog3 functions and targets. (A) Venn diagram showing genes that were upregulated in E15 Neurog3-null cells (yellow) and predicted repressed targets of Neurog3 based on module network analysis in Genomica (orange). Fisher's exact test was used to calculate the P-value. (B). Functional enrichment analysis of biological functions of predicted repressed targets of Neurog3 based on the module network analysis algorithm. (C) Functional gene set analysis of genes that were enriched in Neurog3-null cells vs. E15 Neurog3+ endocrine progenitors (by 2-fold) was performed using DAVID (FDR<0.05), similar biological terms were grouped. (PDF) [file pgen.1004645.s006.pdf]

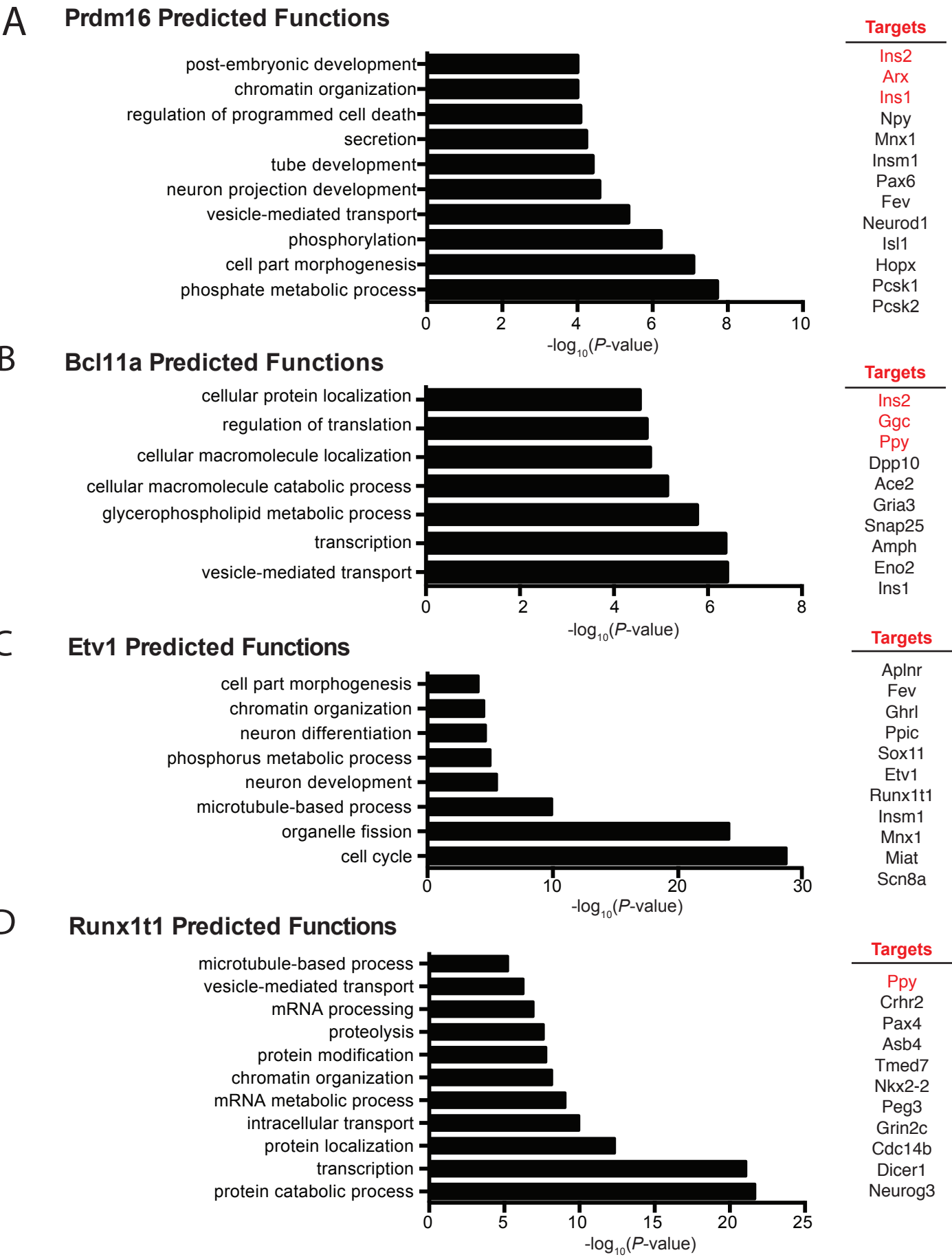

Supplement: Figure S7 — Predicted targets and GO terms of a subset of regulators. (A–D) Predicted biological functions of Bcl11a, Runx1t1, Etv1, and Prdm16 as determined by DAVID analysis of Genomica predicted targets for positively-correlated genes. FDR<0.2. X-axis shows the −log (p-value) of each biological function as calculated in DAVID. A sample of the predicted targets is shown to the right. Validated targets are shown in red. (PDF) [file pgen.1004645.s007.pdf]

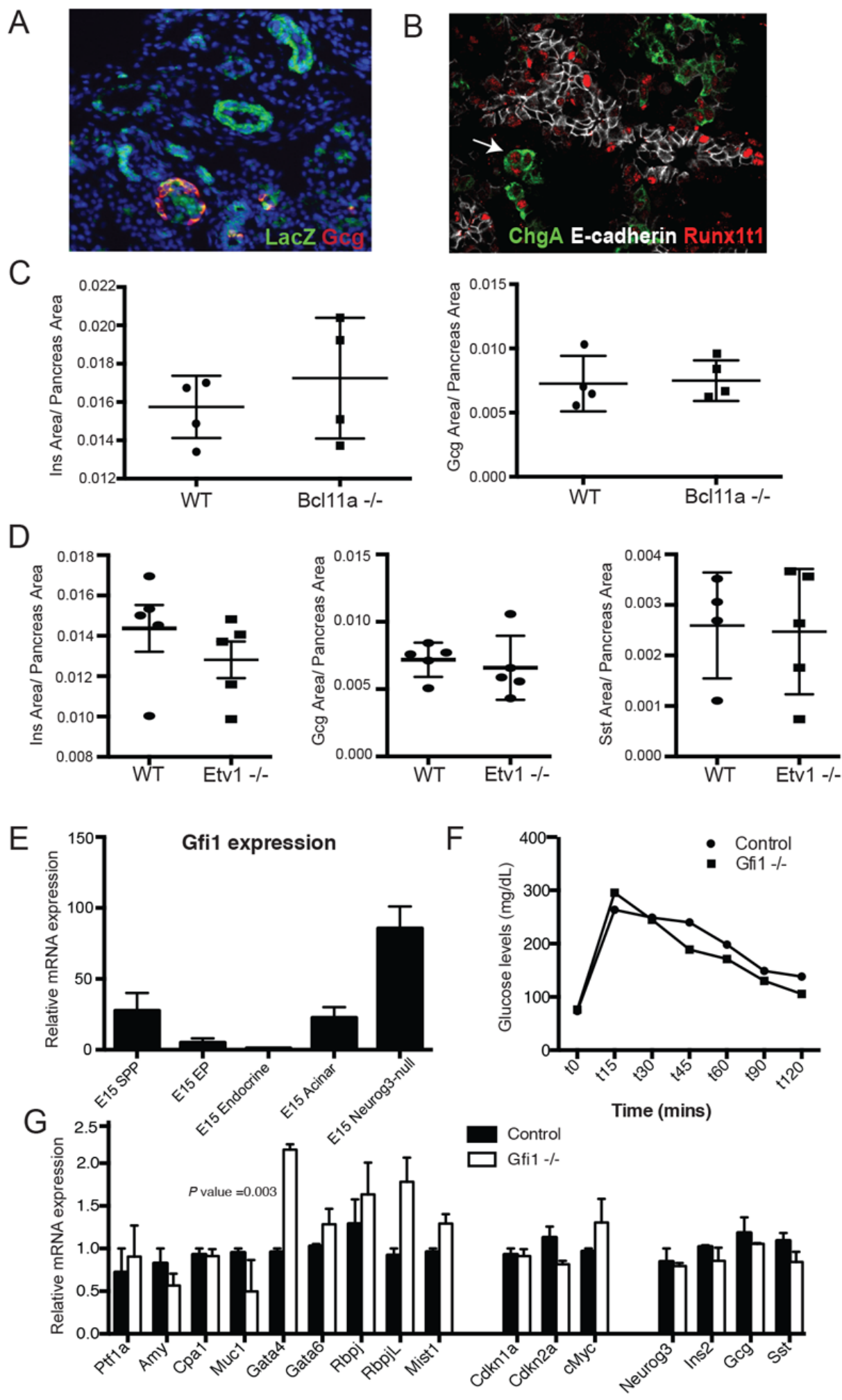

Supplement: Figure S8 — Phenotypic mutant analysis of nominated regulators. (A) Expression of Etv1 expression in adult mouse pancreas using the Etv1LacZ knock-in reporter mouse with Glucagon staining (red). (B) Immunostaining showing that Runx1t1 (green) is expressed in a subset of islets sells as determined by overlap with islet marker Chromogranin A (ChgA, green), epithelial cells are shown in white in E15 fetal pancreas. (C) Morphometric analysis comparing the insulin+ cell area and glucagon+ cell area in Bcl11a mutant mice compared to littermate controls (n = 3 each) at birth (P1). (D) Morphometric analysis of pancreatic polypeptide+ (PP), insulin+ (Ins), and glucagon+ (Gcg), and somatostatin+ (Sst) cell area in Etv1 mutant mice on embryonic day 18 (n = 5, each). In (C) and (D) there were no statistically significant changes in each comparison. (E) mRNA expression of Gfi1 from E15 pancreatic progenitors, E15 endocrine progenitors, E15 endocrine cells, E15 acinar cells, and E15 Neurog3-null cells. (F) Fasting glucose tolerance between 8–12 week old Gfi1 mutant mice and control littermates (n = 3, each). (G) mRNA expression analysis comparing a set of pancreatic markers between Gfi1 mutant whole pancreas and control mice at P1 (n = 2, mean +/− SEM). (PDF) [file pgen.1004645.s008.pdf]
