# Supplementary material for: An Integrated Cell Purification and Genomics Strategy Reveals Multiple Regulators of Pancreas Development
Source: PLoS Genet. 2014 Oct 16;10(10):e1004645. doi: 10.1371/journal.pgen.1004645 (PMC4199491; doi:10.1371/journal.pgen.1004645)
Supplement: Figure S5 — GSEA of various module and iterations parameters used in IMNA. Gene set enrichment analysis displaying the enrichment score and distribution of known regulators of pancreas development based on their frequency. We show 25 modules at 100 iterations, 50 modules at 100 iterations, 75 modules at 120 iterations, and 100 modules at 100 iterations. The enrichment score for these parameters was worse than the enrichment score for 75 modules at 100 iterations. All statistical tests had a P-value and FDR value of <0.05. (PDF) [file pgen.1004645.s005.pdf]

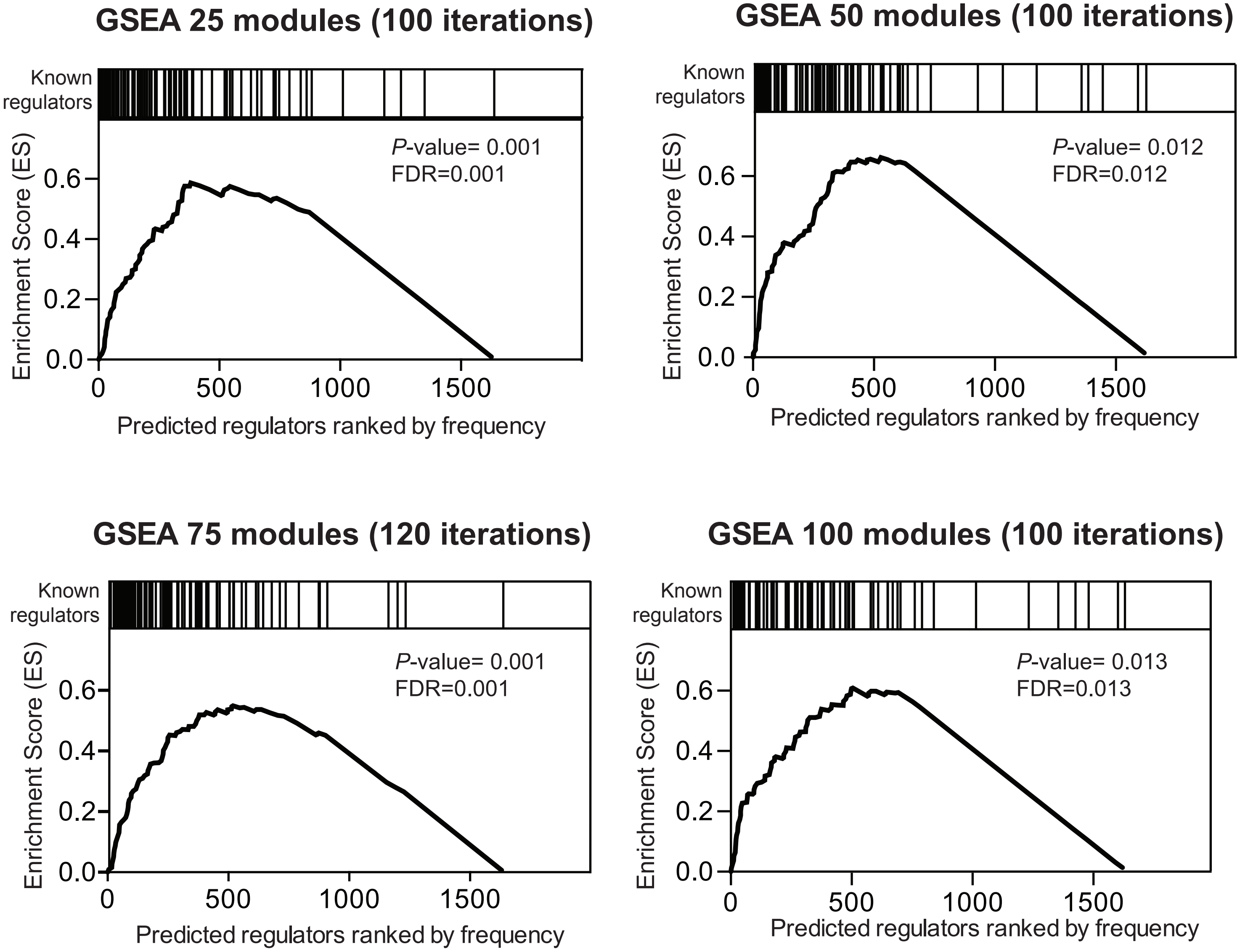
GSEA 25 modules (100 iterations)
Known regulators
Enrichment Score (ES)
P-value= 0.001
FDR=0.001
0.0
0.2
0.4
0.6
0
500
1000
1500
Predicted regulators ranked by frequency
GSEA 50 modules (100 iterations)
Known regulators
Enrichment Score (ES)
P-value= 0.012
FDR=0.012
0.0
0.2
0.4
0.6
0
500
1000
1500
Predicted regulators ranked by frequency
GSEA 75 modules (120 iterations)
Known regulators
Enrichment Score (ES)
P-value= 0.001
FDR=0.001
0.0
0.2
0.4
0.6
0
500
1000
1500
Predicted regulators ranked by frequency
GSEA 100 modules (100 iterations)
Known regulators
Enrichment Score (ES)
P-value= 0.013
FDR=0.013
0.0
0.2
0.4
0.6
0
500
1000
1500
Predicted regulators ranked by frequency
